# Change and convergence of innovation efficiency among listed health companies in China: Empirical study based on the DEA–Malmquist model

**DOI:** 10.3389/fpsyg.2023.1100717

**Published:** 2023-03-09

**Authors:** Yanqi Han, Minghui Hua, Malan Huang, Jin Li, Shixiong Cheng, Xihang Wei

**Affiliations:** School of Business, Hubei University, Wuhan, China

**Keywords:** health industry, innovation efficiency, dynamic transition, convergence trend, DEA-Malmquist

## Abstract

This study investigates the present situation of and changing trend in the innovation efficiency of health industry enterprises in China. Based on panel data for 192 listed health companies in China from 2015 to 2020, we analyse innovation efficiency using the DEA–Malmquist index and test convergence using σ-convergence and β-convergence models. From 2016 to 2019, comprehensive average innovation efficiency increased from 0.6207 to 0.7220 and average innovation efficiency decreased significantly in 2020. The average Malmquist index was 1.072. Innovation efficiency in China as a whole, North China, South China, and Northwest China showed σ-convergence. Except for the Northwest region, absolute β-convergence was evident, and in China as a whole, North China, Northeast China, East China, and South China, conditional β-convergence was evident. Overall innovation efficiency of these companies has increased annually but needs further improvement, and the COVID-19 pandemic has had a great negative impact on it. Innovation efficiency and trends in it vary across regions. Furthermore, we should pay attention to the impacts of innovation infrastructure and government scientific and technological support on innovation efficiency.

## 1. Introduction

Promoting the development of the health industry can help develop healthy human communities and enhance people’s welfare. China issued the outline of the ‘Healthy China 2030’ plan in 2016 (Central Committee of the Communist Party of China, State Council, n.d.), and put forward the ‘Healthy China Action’ in 2019 (State Council, n.d.). These plans raised the creation of a healthier China to the level of national strategy and built a national development model meant to ensure people’s health in a holistic manner. This shows that the health industry has become a new starting point to promote China’s adjustment of industrial structure and promote the scientific development of its economy and society. As China is one of the largest countries in the world, the development of its health industry is of immense significance for achieving the sustainable development goals of the United Nations and promoting coordinated global health governance (Liu et al., 2021). Although the health industry is a ‘sunrise industry’, there are still some problems, such as imperfect policies and regulations, low production efficiency, low degree of information integration (Sisodia and Jindal, 2021), insufficient reserves of compound talent, improper connection of the industrial chain (Pan et al., 2021), and insufficient R&D innovation. The COVID-19 pandemic has highlighted the fundamental role of the health industry in supporting the global health governance system and has accelerated the digital transformation of the entire sector and chain of the health industry. Under strong national policy support and amid the industrial reform led by the new double-cycle pattern (a new development pattern with China’s domestic big cycle as the main body and the mutual promotion of domestic and international double cycles), China’s health industry-related enterprises have mushroomed and grown continuously. Some have achieved outstanding performances in terms of GDP and development scale, and have been listed successfully, becoming key links in the health industry chain. and playing an important leading and exemplary role in promoting the transformation and upgrading of the health industry. Comprehensively considering China’s role in pandemic control and the important position of China’s health industry in the global market, it is evident that improving the innovation efficiency of China’s health industry listed companies not only can provide the impetus to achieve the modernisation of China’s health industry chain and promote the transformation and upgrading of China’s economy but also can promote the construction of healthy human communities.

Therefore, against the international background of global health governance, and the development bottleneck faced by the health industry, it is of great theoretical and practical significance to study the innovation efficiency of Chinese listed health companies quantitatively from the micro perspective of enterprises. To address the deficiencies of existing studies, this study, taking the micro perspective of enterprises, uses 192 Chinese listed health companies from 2015 to 2020 as research samples, constructs an evaluation index system of innovation efficiency, and empirically analyses the innovation efficiency and convergence trend of the companies using the DEA–Malmquist index and convergence model. To enrich theoretical research on health industry innovation efficiency, we then propose an innovation optimisation path for promoting high-quality development in the health industry.

The paper is structured as follows. The second part is a literature review, the third part introduces the data sources and model methods of the article, the fourth part is empirical results, the fifth part is discussion, and the sixth part is conclusions and policy recommendations.

## 2. Literature review

Innovation efficiency has always been the focus of academic research. In recent years, scholars at home and abroad have launched multi-dimensional research around the theme of innovation efficiency. This paper reviews the existing research on the measurement and convergence trend of innovation efficiency. First of all, there are two methods used by scholars to measure the innovation efficiency: stochastic frontier approach (SFA) and data envelopment approach (DEA). The stochastic frontier method (SFA method) is proposed by Aigner et al. (1977) and Meeusen and Julien (1977). This method is mainly applicable to the case of single output. Domestic scholar He (2004) used the SFA method earlier to measure the efficiency of technological innovation; Subsequently, Wang and Peng (2010) added Malmquist method and spatial measurement model to analyze the influencing factors on the efficiency of regional technological innovation based on the SFA method; Scholars such as Xiao et al. (2017) and Chao (2020) use SFA method to analyze the innovation efficiency of green technology and the innovation efficiency of manufacturing industry in China, respectively. Due to the narrow application scope of SFA method and the need to set the function relationship in advance, if the setting of the function relationship is unreasonable, the calculation result is inaccurate. The improved DEA method based on SFA method can solve these problems. American scholars Charnes et al. (1978) proposed this model to compare the relative efficiency between multiple input variables and multiple output variables. At present, scholars also prefer to use DEA method. Nasierowski and Arcelus (2003) used DEA to compare the innovation efficiency between different countries; Zhang et al. (2011) conducted a DEA analysis of innovation efficiency in China region. In the follow-up research, the traditional DEA model needs to be continuously expanded and improved for calculation. For example, Kádárová et al. (2015) combine the DEA method with BSC to establish a comprehensive performance and efficiency management system for industrial enterprises and their processes; Sun and Ma (2019) measured the innovation efficiency of high-tech manufacturing industry in China from the perspective of whole and sub-region by using the method of combining Malmquist model with DEA model. Bresciani et al. (2021) and others used DEA-Bootstrap to measure the innovation efficiency in Italy and Spain. Chen (2022) uses a three-stage DEA model to conduct an empirical study on the innovation efficiency of industrial enterprises in China. Generally speaking, most scholars choose the improved model for DEA method at present. Considering the nature and content of the research object, this paper chooses the dynamic DEA-Malmquist model when measuring the innovation efficiency.

The research on convergence was first carried out in the framework of neoclassical economics. Patel and Pavitt (1994) explored the regional convergence of OCED national innovation efficiency using the σ convergence analysis method. Furman et al. (2002) verified the convergence characteristics of OCED national innovation efficiency with α-convergence and β-convergence analysis. Based on the panel data of 15 European Union countries, Jungmittag (2006) verified the convergence trend of innovation efficiency using the unit root method. However, the traditional panel data model ignores the geographical location of each region or other economic and social factors in the spatial relative relationship, so scholars began to study the spatial factors into the traditional convergence model. Shen et al. (2019) examined the impact of international R&D spillover on the convergence of regional innovation efficiency with a spatial convergence model, and the result showed that it had a promoting effect. Lv et al. (2020) investigated the convergence of green innovation efficiency in 30 provinces of China through α convergence and absolute β convergence and conditional β convergence. Zhang and Guo (2022) used the global Moran index to test the spatial correlation of the technological innovation efficiency of the provincial industry. Zhao et al. (2023) calculated the innovation efficiency of high-tech industries in China and identified the spatial convergence trend using the Markov chain method. In this paper, the spatial factors are also considered when discussing the convergence of innovation efficiency, so as to measure the spatial–temporal characteristics of innovation efficiency of health industries more reasonably.

There are few studies on the overall innovation efficiency of health industry in China and abroad. Most of them study the innovation efficiency of each segment of health industry against the background of health industry. Gascón et al. (2017) and Shin et al. (2018) study the innovation efficiency of pharmaceutical companies as the research object. Kaitelidou et al. (2016) studied the innovation efficiency of public hospitals. Zhang and Yu (2019) studied the innovation efficiency of the medical device industry; Lai and Shi (2021) and Zhong et al. (2022) studied the innovation efficiency of the pharmaceutical industry.

The existing researches on the subject of innovation efficiency and convergence are rich, but there is still room for improvement and deepening. Compared with the existing research, the marginal contribution of this paper may lie in: (1) On the research sample, the current academic research results on enterprise innovation efficiency evaluation are mostly concentrated in industries, high-tech industries, manufacturing and other fields, while the literature on efficiency evaluation of health industries is very scarce. This paper focuses on the field of health industry, combining the definition of health industry by academic circles and the National Bureau of Statistics of China, and defines health industry as “the collection of production activities that provide products (goods and services) directly or closely related to health for the public based on medical and health care, biotechnology and life science, with the purpose of maintaining, improving and promoting the health of the people.” Finally, 192 listed companies in health industry are selected, and the research field of enterprise innovation efficiency is expanded. (2) In terms of research content, there have been many researches on the measurement of innovation efficiency, but most of them are at the industry and regional level, and the research on the convergence of innovation efficiency is still relatively few. This paper analyzes the innovation efficiency at the enterprise level in the health industry from a micro perspective, and further analyzes the industry heterogeneity and spatio-temporal evolution law, which further enriches the research content on the basis of previous research.

## 3. Materials and methods

### 3.1. Data sources

When selecting samples, considering the date when the concept of ‘big health’ was put forward in China (2016) and the date when the outline of ‘Healthy China 2030’ was issued (25 October, 2016), the data sample period of this study was set as 2015–2020. Combined with the Health Industry Statistical Classification (2019), China Securities Regulatory Commission (CSRC) 2012 industry classification in the China Tai’an Database (CSMAR), and current academic research results on health industry classification (Hrazdil et al., 2013; China Statistics Bureau, 2019) the corresponding listed companies were selected. The health industry in the sample includes pharmaceutical manufacturing; health and medical supplies, instruments, and equipment manufacturing; health and medical services; pharmaceutical research and development; pharmaceutical wholesale and retail; and health-related scientific research and technology services. Based on the above background, the original sample was scientifically screened. First, companies whose main business was unrelated to the health industry were eliminated. Second, ST (shares of companies that have suffered losses for two consecutive years, and require special treatment) and *ST (shares of companies that have suffered losses for three consecutive years, and have been given early warning of delisting) shares were excluded, because these companies have abnormal financial situations, which may affect the reliability of the evaluation results. Finally, to ensure the accuracy of the empirical results, this study deleted companies with a large amount of missing input–output index data based on the availability and continuity of various data indicators over time. After screening, 192 listed companies in the health industry with highly relevant main businesses and sound data were sorted. Except for the number of patent applications, taken from the CNRDS database, input–output index data were obtained from the CSMAR database. In addition, in the conditional β-convergence test, the data for the control variables were all from the database of the National Bureau of Statistics of China and the Statistical Yearbook of China.

### 3.2. Selection of indicators

Based on existing research (Haschka and Herwartz, 2020; Liu et al., 2020), the research results of the innovation efficiency evaluation index system, the number of R&D personnel, and the amount of R&D investment were selected as the investment indicators for the innovation efficiency evaluation of listed companies in the health industry. Net profit, operating income, and number of patent applications are output indicators. The specific evaluation index systems are listed in Table 1.

**Table 1:** Evaluation indicators of innovation efficiency of listed companies in the health industry.

Index	Variable name	Indicator description
Input indicator	A1	Number of R&D personnel (person)
	A2	R&D investment amount (yuan)
Output indicator	B1	Net profit (yuan)
	B2	Operating income (yuan)
	B3	Number of patent applications (pieces)

### 3.3. Model approach

This paper uses the research of Fan and Gu (2022) for reference on the measurement method of innovation efficiency, and uses the method of combining DEA with Malmquist model to measure the innovation efficiency of listed companies in health industry in China. In the research on the convergence of innovation efficiency, using the research method of Lv et al. (2020) for reference, the convergence trend of innovation efficiency is studied through σ convergence, absolute β convergence and conditional β convergence. The specific method model is shown below.

#### 3.3.1. DEA–BCC model

The DEA has two basic models: CCR and BCC. Both models are used to measure innovation efficiency when there are multiple inputs and multiple outputs. However, CCR measures the relative efficiency of input and output under the condition of constant return to scale while BCC under changing return. Since the innovation and development of the health industry involves complex and systematic behaviour with uncertain marginal returns (Lin and Luan, 2020), this study introduces the output-oriented DEA–BCC model under the condition of a variable scale to measure and evaluate the innovation efficiency of Chinese listed health companies from 2016 to 2020 (because this study carries out a lag process on the data when measuring innovation efficiency, and thus, the sample data start from 2015). The basic form of the model is as follows:


$$\min\;\theta =\left[\theta_{0}-\varepsilon \left(\sum_{i=1}^{m}s_{r}^{+}+\sum_{i=1}^{m}s_{r}^{-}\right)\right]$$

$$s.t.\overset{n}{\underset{j=1}{\sum }}\lambda_{j}X_{ij}+S_{i}^{-}=\theta_{0}X_{\mathrm{io}}$$

$$\overset{n}{\underset{j=1}{\sum }}\lambda_{j}y_{ij}-s_{i}^{+}=y_{r0}$$

$$\overset{n}{\underset{j=1}{\sum }}\lambda_{j}=1$$

$$\lambda_{j}\geq 0,s_{r}^{+}\geq 0,S_{i}^{-}\geq 0,i=1,2,\ldots ,m$$

(1) r=1,2,…,s,j=1,2,…,n,

where θ is the DEA pure technical efficiency score of the DMU, which ranges from 0 to 1.In this paper, it represents the measured innovation efficiency, which is the variable that this paper focuses on; si + indicates insufficient output; and si-indicates input redundancy. When θ is 1 and si + = si- =0, the decision-making unit is a strong effective unit; when si + and si-are not equal to 0, the decision-making unit is weak effective unit, which may have problems of input redundancy and uneven resource allocation; when the value of θ is less than 1, it indicates that the decision-making unit is in an invalid state, and the efficiency of resource allocation should be improved.

#### 3.3.2. Malmquist index

The analysis of static DEA-BCC model is based on the fact that the production technology remains unchanged during an investigation period. In order to carefully investigate the dynamic change of innovation efficiency of engraving under the condition that the production technology changes from 2016 to 2020, and better adapt to the panel data, it is necessary to further introduce Malmquist index of R&D and innovation. The Malmquist index, proposed by Malmquist (1953), is a dynamic analysis method based on the DEA model. Based on a combination of this theory and DEA theory, scholars such as Fare have expanded the application range of the Malmquist index in various fields (Ray and Desli, 1997). The Malmquist index can be divided into technical efficiency (EC) and technical progress (TC), and the relationship is


(2) $${MI}_{\mathrm{it}}={EC}_{\mathrm{it}}\times {TC}_{\mathrm{it}}\text{,}$$

where EC represents the movement of the production frontier from period t to period *t* + 1, and TCit represents the catch-up speed of a certain DMU to the production possibility boundary from period t to period t + 1. When the Malmquist index is greater than 1, it indicates that the total efficiency shows an upward trend with the passage of time; when it is equal to 1, it means that the total efficiency does not change with time; when less than 1, it shows a downward trend. When the increase in the level of productivity; otherwise, there will be a tendency for the level of productivity to decrease. In this paper, the overall trend of innovation efficiency of the company in the past 5 years is judged by the size of MI index.

#### 3.3.3. σ-Convergence

This study uses σ-convergence to test whether the degree of dispersion of innovation efficiency of listed health industry companies in different regions decreases over time. According to Barro and Sala-i-Martin (1992), if the standard deviation of innovation efficiency in different regions decreases with time, innovation efficiency shows σ-convergence. In the calculation process, the standard deviation of the logarithm of innovation efficiency is usually adopted, as shown in Equation (3), where $\mathrm{IN}N_{i,t}$ is the innovation efficiency of region i in period t, $\mathrm{lnIN}N_{i,t}$ is the log value of the scientific and technological innovation efficiency of region i in period t, and N is the number of regions.


(3) $$\sigma_{t}=\sqrt{\underset{i}{\sum }{\left(\mathrm{lnIN}N_{i,t}-\mathrm{lnIN}N_{t}\right)}^{2}/N}\text{.}$$

#### 3.3.4. β-Convergence

β-convergence is the most commonly used method in convergence models, including absolute and conditional β-convergence. Absolute β-convergence is a trend of convergence between regions without considering the influence of external factors, whereas conditional β-convergence is a trend of convergence between regions considering external factors. Drawing on the demonstration method of Barro and Sala-i-Martin (1992), the traditional absolute β-convergence model has the form:


(4) $$\ln\left(\frac{\mathrm{IN}N_{i,t+1}}{\mathrm{IN}N_{i,t}}\right)=\alpha +\beta ln\left(\mathrm{IN}N_{i,t}\right)+\mu_{i,t}\text{,}$$

where ln,.($\mathrm{IN}N_{i,t+1}$/$\mathrm{IN}N_{i,t}$) is the growth rate of innovation efficiency in period t of region i; $\mathrm{IN}N_{i,t+1}$ is the innovation efficiency of region i in period t + 1; $\mathrm{IN}N_{i,t}$ is the innovation efficiency of region i in period t; μ i,t is the error term; and β is the parameter to be estimated. If β is less than 0, it indicates that there is a convergence trend in innovation efficiency; if β is greater than 0, it indicates that there is a divergent trend in innovation efficiency. $\beta =-\left(1-e-\lambda T\right)/T$ λ is the convergence speed of β-convergence, $\lambda =-\ln\left(1-||\beta ||\right)/T$. Absolute β-convergence means that the efficiency of innovation in an inefficient area grows faster than in a high-efficiency area. In actual analysis, some scholars have found multiple equilibria, and established that regions with large structural differences tend to have different equilibrium points. To this end, Barro (1991) proposed conditional convergence, which was extended by Barro and Sala-i-Martin (1992) and Mankiw et al. (1992), among others. Compared with absolute β-convergence, conditional β-convergence considers that the growth rate of efficiency depends not only on the initial efficiency level but also on factors. Conditional β-convergence implies that different regions converge to their own steady state. Because of the differences in the level of economic development, government scientific and technological support, and innovation infrastructure in different regions, this study draws on the research of Zhang and Guo (2022) and other research, adds these control variables to the absolute convergence test model, and constructs a conditional convergence test model, as shown in Formula (5).


(5) $$\ln\left(\frac{\mathrm{IN}N_{i,t+1}}{\mathrm{IN}N_{i,t}}\right)=\alpha +\beta ln\left(\mathrm{IN}N_{i,t}\right)+\mathrm{\mu lnA}\text{.}$$

In Formula (5), A is the control variable and μ is the regression coefficient of the control variable. If the regression coefficient of the control variable is positive, then the corresponding control variable is an improvement factor for innovation efficiency. Economic development indicators are measured by *per capita* real GDP, innovation infrastructure indicators are measured by the proportion of total telecommunications business to GDP, and government science and technology support is expressed by the proportion of local financial technology expenditure in the general budget expenditure of the local government. The data in the model are represented by the logarithm of the original data, to alleviate the heteroscedasticity of the model.

## 4. Results

### 4.1. Data pre-processing

To improve the reliability of the data analysis results, the following three pre-processing steps for the collected sample data were carried out before the empirical analysis.

- Missing data processing. To ensure the integrity of the data, this study first deletes a large number of missing company samples of input–output index data; for patent application data, this study matches the corresponding data in the CNRDS database according to the stock code. On this basis, this study combined the annual reports of listed companies and the Juchao Information Network to fill in missing data.
- Lag period processing. Because of the time lag between the innovation input and innovation output of listed companies in the health industry, in order to more accurately reflect innovation efficiency, this study draws on the research of previous scholars and sets the input–output time lag to 1 year; that is, the input index measurement period is 2015–2019, while the output indicator measurement period is 2016–2020.
- Dimensionless processing. On the one hand, because the DEA model can only identify non-negative data in the calculation process, there were a small number of negative numbers in the original data on net profit and operating income in this study. However, there was a large difference between the values of different indicators in the original data used in this study. If calculated directly, the calculation results might then have been inaccurate if the effect of the decimal values were ignored. Considering these two factors, this study normalises the original data; the processing formula is

(6) $$X^{*}=0.1+0.9\times \frac{X_{i}-\min\left(X_{i}\right)}{\max\left(X_{i}\right)-\min\left(X_{i}\right)}\text{,}$$

where X* is the normalised data, and Xi is the original data. After dimensionless processing, all values are distributed between [0.1, 1], the values are only translated or scaled, and the shape of the production front surface does not change, ensuring that the data conform to the operation rules and will not affect the research result.

### 4.2. Basic descriptive statistics of sample data

#### 4.2.1. Statistics by region

This study counts the regional distribution of 192 listed companies in the health industry according to the provinces where the sample companies are located and then the seven administrative geographical divisions of the country (containing the provinces). Among the selected samples, the number of listed companies in the health industry is the largest in East China and the smallest in Northwest China, showing spatial distribution characteristics of decreasing from east to west and from south to north. The number of distributions in each region was 71 in East China, 34 in North China, 29 in South China, 24 in Southwest China, 17 in Central China, 13 in Northeast China, and 4 in Northwest China, accounting for 36.9, 17.7, 15.1, 12.5, 8.8, 6.7, and 2%, respectively. In terms of the number of listed companies in the health industry by province and city, Zhejiang (25), Guangdong (22), and Beijing (16) have the most, whereas Liaoning (1), Gansu (1), and Xinjiang (1) have the least. The details of the number of companies listed in the health industry in each province are in Table 2.

**Table 2:** Number of listed companies in the health industry by province.

East China	North China	South China	Southwest China	Central China	Northeast China	Northwest China
Zhejiang 25	Beijing 16	Guangdong 22	Chongqing 7	Hunan 8	Jilin 8	Shanxi 2
Jiangsu 13	Tianjin 7	Hainan 4	Sichuan 5	Hubei 5	Heilongjiang 4	Gansu 1
Shanghai 12	Shanxi 5	Guangxi 3	Yunnan 4	Henan 4	Liaoning 1	Xinjiang 1
Shandong 10	Hebei 3		Guizhou 4			
Jiangxi 5	Inner Mongolia 3		Tibet 4			
Anhui 4						
Fujian 2						
Total 71	Total 34	Total 29	Total 24	Total 17	Total 13	Total 4

#### 4.2.2. Statistics by industry classification

**Figure 1:**
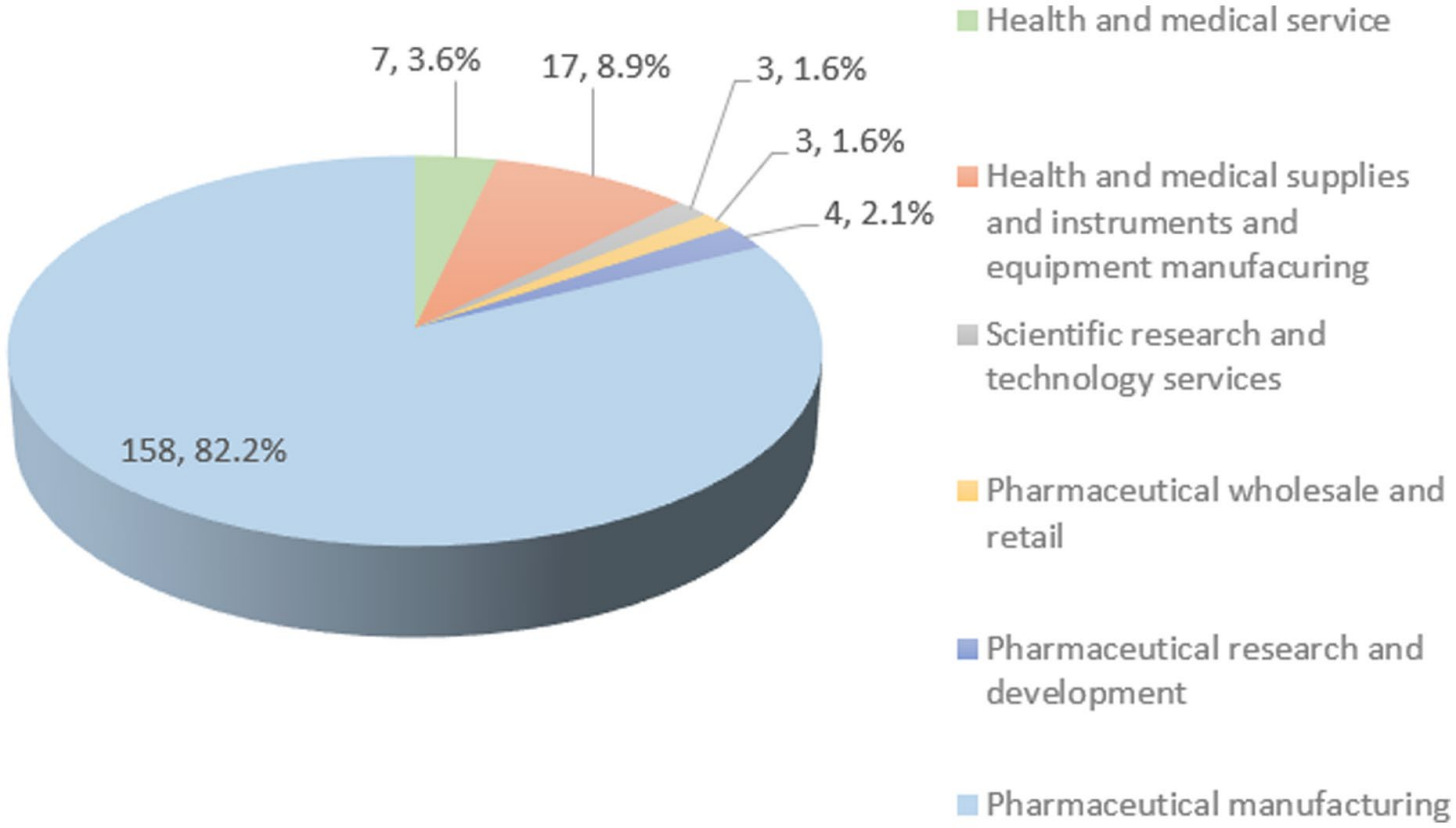
Industry distribution of 192 listed companies in the health industry in China, 2015–2020.

Based on the Health Industry Statistical Classification (2019), China Securities Regulatory Commission (CSRC) 2012 industry classification in the China Tai’an Database (CSMAR), and the main business of each company, the 192 listed health companies in the sample were classified into pharmaceutical manufacturing; health and medical supplies, instruments, and equipment manufacturing; health and medical services; pharmaceutical research and development; pharmaceutical wholesale and retail; and health-related scientific research and technology services. Pharmaceutical manufacturing refers to the process by which raw materials become new pharmaceutical products after physical or chemical changes, including what is commonly referred to as the manufacturing of Chinese and Western medicines. The manufacturing of health and medical supplies and instruments refers to the production and manufacture of instruments, equipment, instruments, *in vitro* diagnostic reagents, calibrators, materials, and other similar or related articles that are directly or indirectly used in the human body. The health and medical services industry includes related healthcare items such as healthcare, rehabilitation, medical care, health consultation, investment and construction of medical and health institutions, and many other fields. The pharmaceutical research and development industry and pharmaceutical wholesale and retail industry are easy to understand, and are not explained here. Scientific research and technology services refers to technology development, transfer, consultation, and technical services in the fields of health science and technology. Among the 192 listed health companies in this study, the pharmaceutical manufacturing industry accounts for the majority. The specific distributions are shown in Figure 1. The first part of Figure 1 represents the number of companies in this segment and the second part represents the proportion of the number of companies in this segment to the total number of companies in the whole health sector.

**Figure 2:**
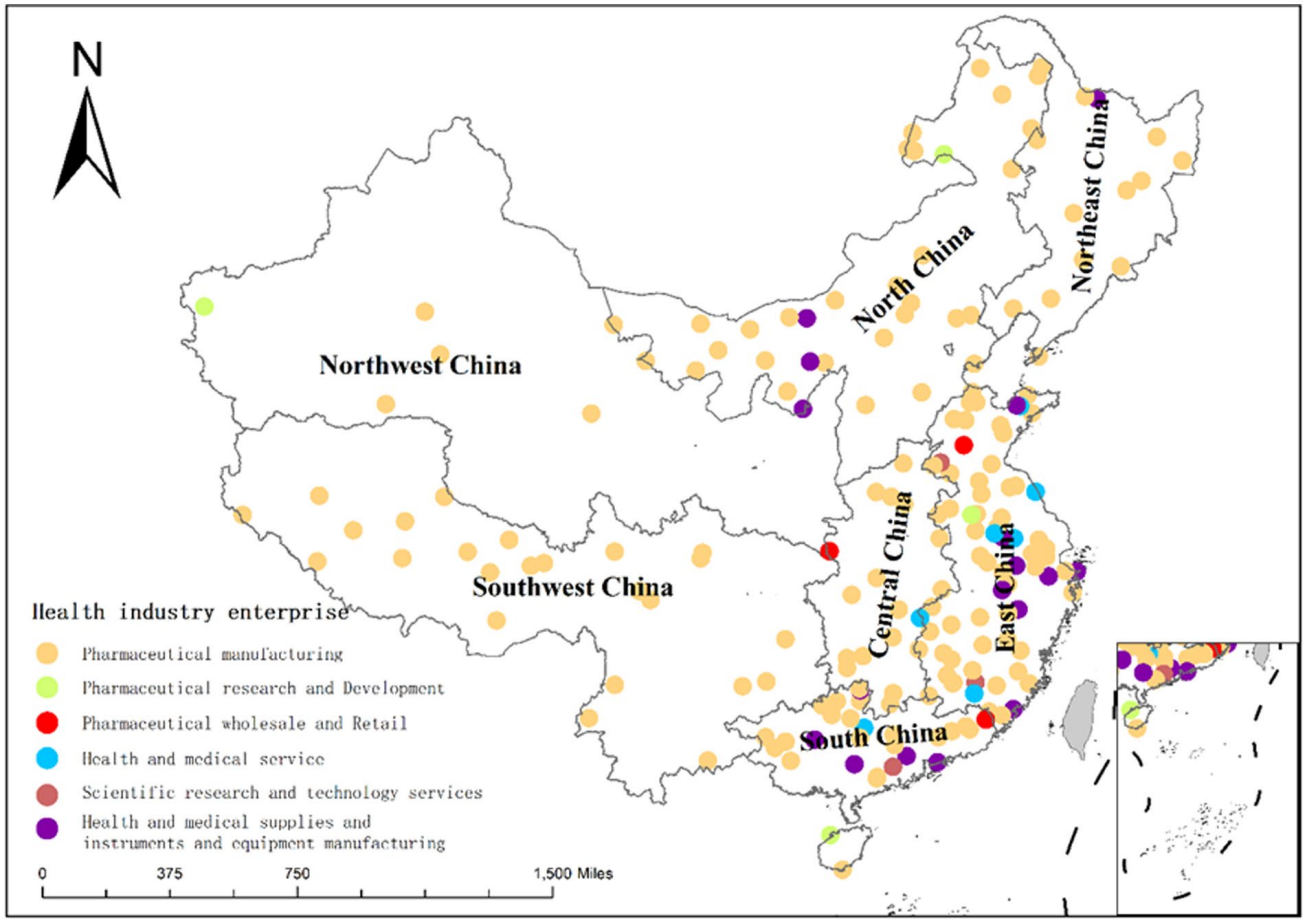
Distribution of the number of listed companies in the health industry by industry by region.

To visualise the spatial distribution characteristics of listed health companies in different subsectors across the seven regions of China, this study uses ArcGIS10.2 software. As shown in Figure 2, the spatial clustering status of listed health industry companies in China is gradually shifting from west to east, from an east-centred distribution to an even more east-centred distribution. The distribution of health industry listed companies in East China is the most intensive, while that in Northwest China is relatively sparse. In terms of industry distribution, the pharmaceutical manufacturing industry accounts for an absolute majority, and its distribution is very wide, covering all seven regions. Health and medical supplies, instruments, and equipment manufacturing companies are relatively concentrated in South, East, and North China. The number of companies in other industries is small, and their distribution is scattered.

### 4.3. The spatial–temporal transition of innovation efficiency of listed companies in the health industry

#### 4.3.1. Static analysis

DEAP 2.1 software was used in this study to analyse the innovation efficiency of listed health industry companies from 2016 to 2020 comprehensively. The innovation efficiency values of the 192 listed health industry companies are shown in Appendix A. This study takes China’s seven administrative geographic regions as a frame to measure and report the average value of comprehensive innovation efficiency in each region from 2016 to 2020. For more details, see Table 3.

**Table 3:** Average innovation efficiency of listed companies in health industry divided by region.

Region	2016	2017	2018	2019	2020	Mean
Northeast China	0.5637	0.5754	0.6568	0.6541	0.5488	0.5998
North China	0.5484	0.5760	0.6280	0.6928	0.5368	0.5964
East China	0.5833	0.6027	0.6367	0.7066	0.5926	0.6244
South China	0.5590	0.6154	0.6574	0.7302	0.6024	0.6329
Central China	0.6238	0.5911	0.6629	0.7394	0.5745	0.6384
Northwest China	0.8693	0.8248	0.6975	0.7833	0.5878	0.7525
Southwest China	0.5975	0.6116	0.6563	0.7476	0.5902	0.6407
Mean	0.6207	0.6281	0.6565	0.7220	0.5761	0.6407

Overall, from 2016 to 2019, the comprehensive average innovation efficiency of listed health industry companies across the seven regions increased from 0.6207 to 0.7220, showing an upward trend year by year. However, the overall efficiency value did not exceed 1, indicating that the innovation development level and resource allocation efficiency of listed health industry companies in each region still have room for improvement. Indeed, in 2020, the average innovation efficiency of each region showed a significant decline, which may have been due to the COVID-19 pandemic resulting in a surge in social demand for medical and health products. At this stage, Chinese listed health industry companies focus more on production, manufacturing, and retail rather than technological innovation and research and development. Consequently, resource allocation of the health industry in each region changes in 2020, and average innovation efficiency declines.

Locally, the average innovation efficiency of listed health industry companies in different regions of China generally increase yearly from 2016 to 2019 and decrease from 2019 to 2020, in the following spatial order of efficiency: Northwest China > Southwest China > Central China > South China > East China > Northeast China > North China. Innovation efficiency gradually decreases from west to east and from south to north, which indicates that although the East region has advantages in innovation resources, such as talent and technology, these resource elements are not fully allocated and utilised, and innovation input and innovation output do not reach ideal expectations.

#### 4.3.2. Dynamic analysis

Using DEAP 2.1 software and the DEA–Malmquist model, this study explores the innovation efficiency of listed companies in the health industry in China from a dynamic perspective from 2016 to 2020, analyses the development trend of innovation efficiency in the calculation period, and calculates the determinants of total factor productivity growth. The decomposition quantity of the Malmquist index is calculated, and the data of the innovation efficiency Malmquist index and decomposition quantity of 192 listed companies are shown in Appendix B. The Malmquist index annual average summary results are shown in Table 4.

**Table 4:** Annual average Malmquist index.

Year	Technical efficiency	Technical progress	Pure technical efficiency	Scale efficiency	Malmquist index
2016–2017	1.04	1.049	1.016	1.023	1.091
2017–2018	1.079	1.435	1.134	0.951	1.548
2018–2019	1.105	0.947	1.075	1.028	1.047
2019–2020	0.809	0.923	0.851	0.95	0.746
Mean	1.001	1.071	1.013	0.988	1.072

As shown in Table 4, overall, the average TFP of listed companies in China’s health industry is 1.072, showing a relatively stable trend of growth in innovation efficiency. The Malmquist index is then decomposed and expanded: the average technical efficiency is 1.001, indicating that effective management methods and decision-making methods promote the improvement of technical efficiency; the average technical progress is 1.071, that is, the growth rate is 7.1%, indicating that the technical level is gradually improving; the average pure technical efficiency is 1.013, indicating that the application of technology is constantly improving; and the scale efficiency is 0.988, indicating that the scale has not reached the optimum. Technological progress and the growth of technical efficiency together contributed to the improvement in overall efficiency, with a growth rate of 7.2%.

**Figure 3:**
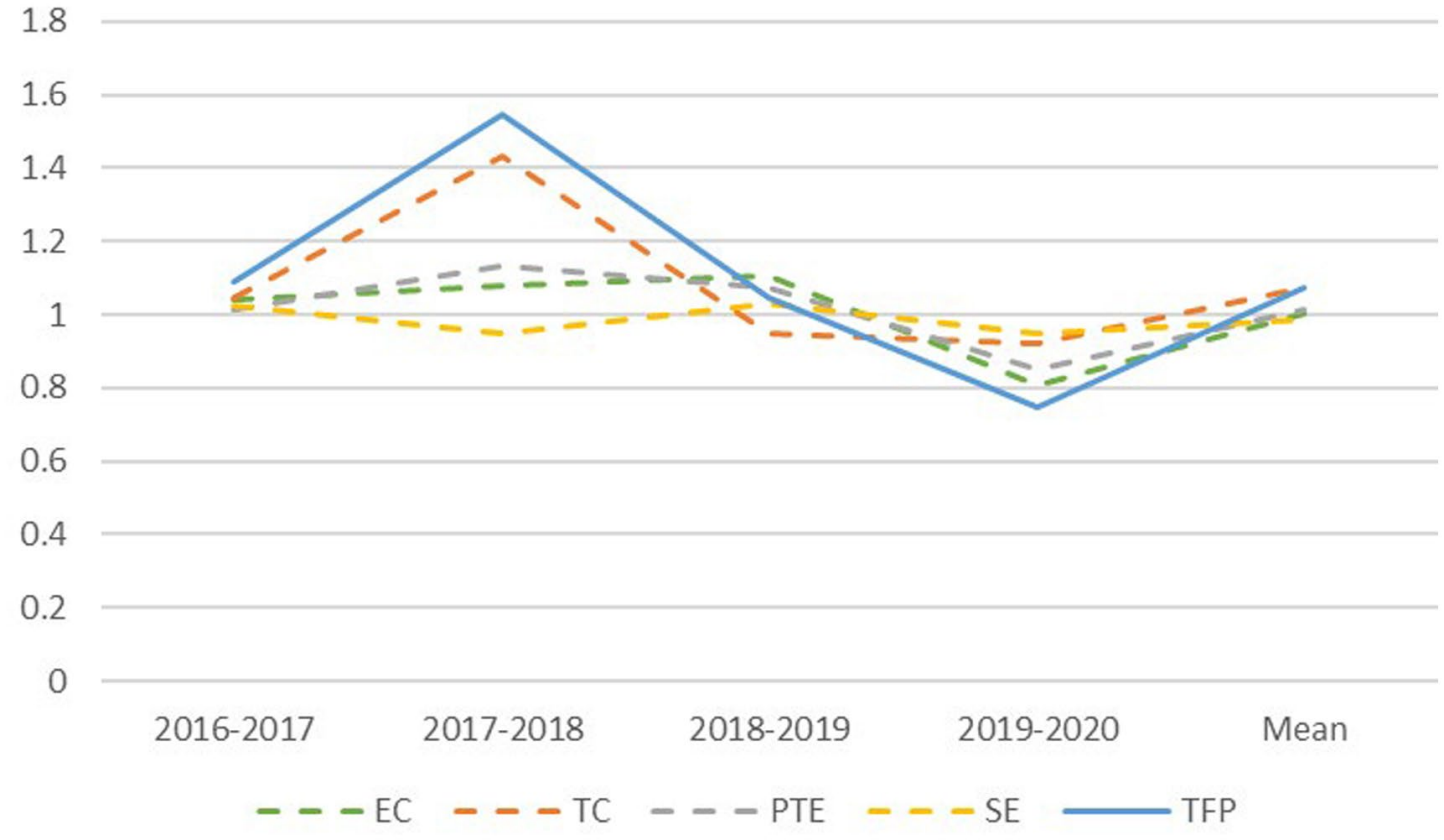
Change trend of annual average of Malmquist index.

As shown in Figure 3, from 2016 to 2020, the overall trend of change in innovation efficiency on the Malmquist index of listed companies in China’s health industry was unstable, as the development trend during the measurement period first decreased and then increased. The figure clearly shows that the change trends of the Malmquist index and technological progress index remain consistent, while the values of comprehensive technical efficiency, pure technical efficiency and scale efficiency fluctuate around 1, maintaining relative stability. This shows that the Malmquist index growth of innovation efficiency is determined to a large extent by the changes in technological progress.

### 4.4. Listed health companies

Listed companies in the health industry have different business objectives and products due to their different industrial subsectors, so there are differences in the input of innovation resources for each company and in each company’s output. The analysis of heterogeneity by subsector in the innovation efficiency of listed health companies is helpful in implementing efficiency improvement strategies tailored to local conditions. Based on the previous industry classification of listed companies in the health industry, this study calculated the overall average value of innovation efficiency in six industries from 2016 to 2020. Further details are provided in Table 5.

**Table 5:** The average innovation efficiency of listed companies in the health field by industry.

Industry	2016	2017	2018	2019	2020	Mean
Pharmaceutical manufacturing	0.5787	0.5928	0.6342	0.7119	0.5658	0.6167
Pharmaceutical research and development	0.66	0.8078	0.7725	0.709	0.701	0.7301
Pharmaceutical wholesale and retail	0.8127	0.7907	0.8753	0.8507	0.8617	0.8382
Scientific research and technology services	0.548	0.5147	0.5653	0.6493	0.5663	0.5687
Health and medical supplies and instruments and equipment manufacturing	0.5574	0.59	0.659	0.72	0.6058	0.6264
Health and medical service	0.627	0.6977	0.7357	0.7131	0.6333	0.6814
Mean	0.6306	0.6656	0.707	0.7257	0.6556	0.6769

Overall, the average innovation efficiency of the six health subsectors in China showed change over time, increasing from 2016 to 2019 and decreasing from 2019 to 2020. The average five-year overall innovation efficiency across the six industries is 0.6769, which is low. This means that there is still much room for improvement in resource allocation and utilisation efficiency. From the perspective of subsectors, from 2016 to 2020, except for the overall increase in the innovation efficiency value of the pharmaceutical wholesale and retail industry, other health industry subsectors showed a trend of increase first and then decrease. The order of average values for each industry is as follows: pharmaceutical wholesale and retail > pharmaceutical research and development > health and medical services > health and medical supplies and instruments and equipment > pharmaceutical manufacturing > scientific research and technology services. The average innovation efficiency of the pharmaceutical wholesale and retail, pharmaceutical research and development, and healthcare service industries is higher than the national average, while other industries are lower than the average. This shows that besides traditional medicine manufacturing, the medical service industry is booming in China’s health industry, which reflects the increasing demand of Chinese citizens for more targeted health services.

### 4.5. Convergent tendency of innovation efficiency in listed health companies

#### 4.5.1. σ-Convergence test

**Figure 4:**
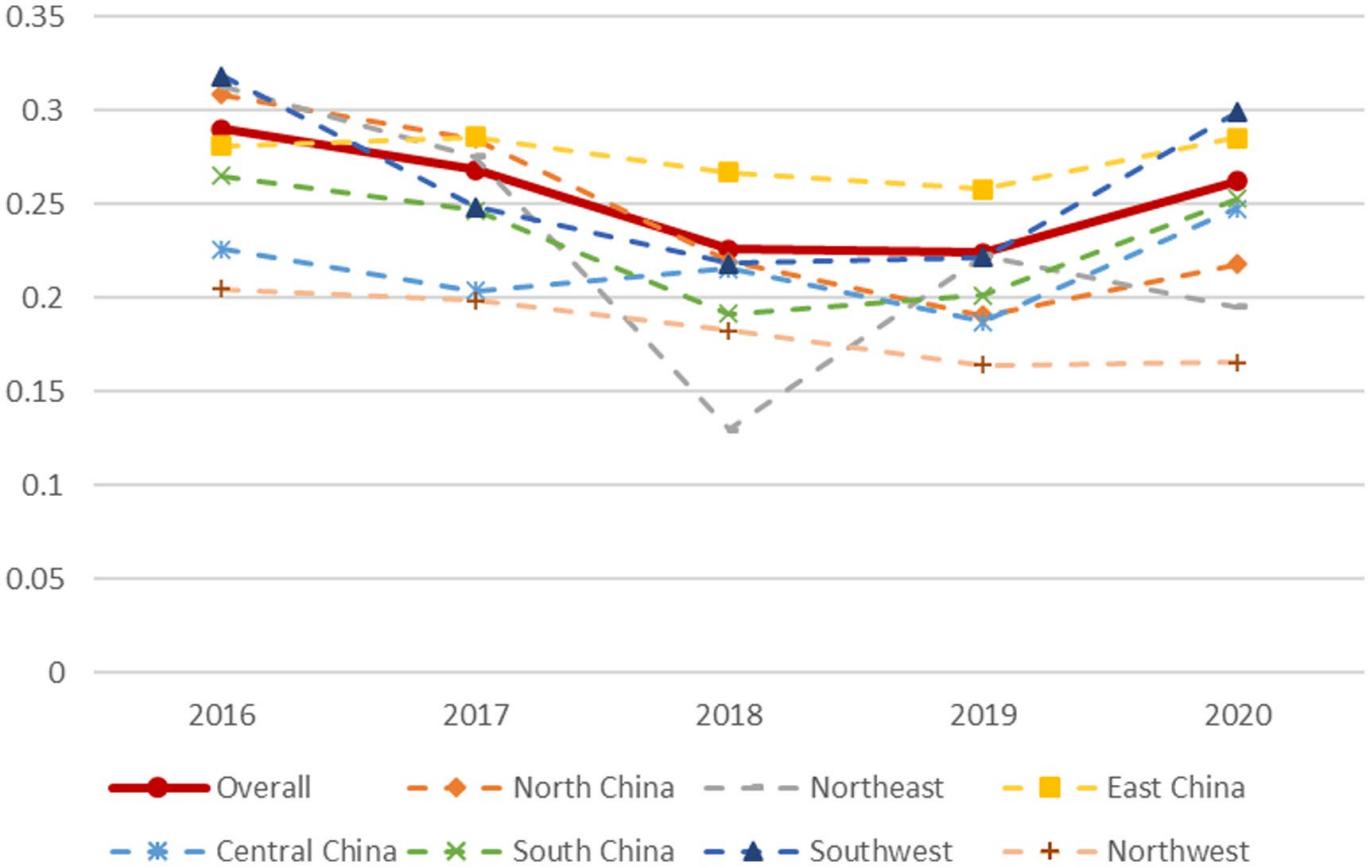
Changes in the σ value of innovation efficiency of listed companies in the health industry in various regions.

Using Formula (3), this study analysed the innovation efficiency of listed health industry companies in China as a whole and in each of seven administrative geographical divisions from 2016 to 2020. To more intuitively describe the evolution of the σ-convergence coefficient of the innovation efficiency of listed health companies across the country and these regions, as shown in Figure 4, a polyline figure is drawn based on the results of the σ-convergence tests on innovation efficiency in each region. Overall, the national innovation efficiency σ-coefficient shows a downward trend, with 2019 as the inflection point; that is, it rebounds slightly from 2019 to 2020. This shows that the overall trend in innovation efficiency among listed companies in the Chinese health industry is σ-convergence and that its degree of dispersion decreases over time. The innovation efficiency of listed companies in the health industry in North China and Northwest China σ-convergence is similar to that of the whole country, with 2019 as the inflection point, showing a trend of convergence before divergence. South China and Southwest China took 2018 as an inflection point, first converging and then diverging. Northeast China, East China, and Central China have fluctuated many times in 5 years, and it can be judged that there is no σ-convergence in these areas. In summary, the deviation in innovation efficiency among listed health companies across China and in North China, South China, and Northwest China shows a gradually narrowing trend; however, there is still a large gap in innovation efficiency of listed companies in Northeast China, East China, and Central China. The reasons for this difference can be further explored in future studies.

#### 4.5.2. β-Convergence test

Based on Equations (4) and (5), the convergence of the β-coefficient of the innovative efficiency of listed companies in various regions is measured. Table 6 reports the test results for absolute and conditional β-convergence. Overall, the absolute convergence of the β-coefficient of innovation efficiency across the country is less than 0, which passes the significance test at the 1% level. Geographically, the β-coefficients of six administrative regions (that is, all of them other than the Northwest region) are significantly negative, indicating that not only the whole country but also most regions show absolute convergence of β. In other words, the innovation efficiency level of the whole country, North China, Northeast China, East China, Central China, South China, and Southwest China will converge to their respective steady state over time. In terms of convergence rate, for the whole country and North China, Northeast China, East China, Central China, South China, and Southwest China it is 0.15, 0.16, 0.68, 0.11, 0.19, 0.14, and 0.15, respectively. In other words, the convergence rate shows the characteristics of Northeast China > Central China > North China > National China = Southwest China > South China > East China.

**Table 6:** Test results for absolute convergence and conditional convergence of β.

Regression coefficient	Overall	North China	Northeast China	East China
Absolute β-convergence coefficient	−0.5314109*** (0.0587616)	−0.5422236*** (0.1033124)	−0.9665268*** (0.2438458)	−0.405928*** (0.094245)
Conditional β-convergence coefficient	−0.5249996*** (0.0595692)	−0.5603628*** (0.1128702)	−0.217712*** (0.193998)	−0.401621*** (0.092205)
Economic level	0.0745072** (0.0628417)	0.1207163*** (0.1379149)	12.88236*** (3.753764)	−0.0907269** (0.0912332)
Government support	0.0089906* (0.0112441)	−0.030131 (0.0685764)	−11.41522*** (2.857796)	−0.0053123 (0.0100423)
Entrepreneurship foundation	0.0963243** (0.0879291)	0.0549212 (0.088212)	−0.1950902* (0.0968124)	0.1495513*** (0.115778)
**Regression coefficient**	**Central China**	**South China**	**Southwest China**	**Northwest China**
Absolute β-convergence coefficient	−0.6178154** (0.2452894)	−0.4999705*** (0.1401917)	−0.5175176** (0.2422383)	−0.026545 (0.4379196)
Conditional β-convergence coefficient	−0.5682027 (0.4481691)	−0.4579644*** (0.1320543)	−0.4900153 (0.2866448)	−0.34155 (0.4710108)
Economic level	1.228266 (2.58497)	−8.221048* (36.01217)	0.1177364 (0.3768255)	−1.34155 (−0.4710108)
Government support	−0.7102414 (2.46856)	3.522766** (15.19719)	−0.0752514 (0.0751906)	−1.212328 (0.8235388)
Entrepreneurship foundation	1.915429 (2.006568)	−1.157541* (0.5745563)	0.0455 (1.125732)	−1.693269 (21.02671)

The β-coefficient of conditional convergence of innovation efficiency in North China, Northeast China, East China, and South China is significantly negative. This shows that considering the three control variables of economic development level, government science and technology support, and innovation infrastructure, the innovation efficiency of listed companies in the health industry in the whole country and in the above four regions will eventually converge to their own steady state over time. The convergence rates of the whole country, North China, Northeast China, East China, and South China were 0.15, 0.16, 0.05, 0.10, and 0.12, respectively, showing the characteristics of North China > National China > South China > East China > Northeast China. After the introduction of control variables, the convergence rate of Northeast China changes greatly, which shows that economic level, government support, and innovation infrastructure have an especially large influence on the growth rate of innovation efficiency among listed companies in Northeast China’s health industry. From the perspective of the influencing factors, the national economic level, government support, and innovation infrastructure all have a positive effect on the value added by listed health companies. At the regional level, the impact of economic level is significantly negative, which indicates that although some regions with higher economic development levels have higher innovation efficiency, their innovation efficiency growth rate is lower than that of regions with lower economic level (Lv et al., 2020). For Northeast China and South China, more attention should be paid to the impact of government support and innovation infrastructure on the innovation efficiency of listed health companies.

## 5. Discussion

The innovation efficiency of the listed companies in the health industry in China has not yet exceeded 1. According to the current development situation of the health industry in China, China’s attention and investment in the health industry are far from enough when compared with those in developed countries. The health industry is still in its infancy, so the companies in this industry still need some time to mature. However, from a dynamic point of view, the overall innovation efficiency is increasing, and technological progress will significantly promote the improvement of enterprise innovation efficiency. This indicates that China’s promotion policy for the health industry is taking effect, and the future investment of resources can be appropriately tilted towards technology support and innovation. The research on health industry in China is still in the initial stage, and no unified definition has been formed for health industry. This paper defines the concept of health industry based on the national level and the common views of the academic community, and classifies the industries according to the sample situation. From the performance of each industry, the innovation efficiency of medical service and medical R&D is higher than that of traditional medical equipment and medical manufacturing, which indicates that the health industry in China is gradually changing from traditional manufacturing to health service, paying more attention to drug R&D, and also reflects the changes in national health demand in China. From the convergence results of this paper, except for the northwest region, the enterprises with low innovation efficiency in other regions of the country have obvious catching-up effect. The gap between them and the advanced enterprises gradually narrows, and eventually tends to the same steady-state level. It shows that although the health industry in China started late, its development speed is fast, and there is a large room for enterprises to rise. Due to the low concentration of industries in the northwest region, the convergence trend is not significant.

Domestic scholars such as Lai and Shi (2021) have studied the innovation efficiency of the pharmaceutical manufacturing industry in the health industry in China. The research conclusion is similar to the research result of this paper to a certain extent, that is, there is spatial heterogeneity in the innovation efficiency of the listed companies in the health industry, and the innovation efficiency level in the southern region is higher than that in the northern region. Therefore, it can be seen that the southern region of China has more advantages in geographical location and economic basis than the northern region, and it is easier to drive the development of enterprises. However, in the research on the value of innovation efficiency in the east and the west, this paper is contrary to the conclusions of Lai Hongbo. Through empirical analysis, this paper finds that the innovation efficiency level in the western region is higher than that in the eastern region. The reason for this difference may be that Lai Hongbo’ research object is smaller than this paper, and they only study the pharmaceutical manufacturing industry in the health industry, and because of the availability of data, the values of western regions such as Tibet are excluded, resulting in the difference in research results. The economic bases of the eastern and western regions differ greatly. Compared with the western region, the eastern region has more concentrated personnel and better infrastructure, but its innovation efficiency is lower. It may be that natural advantages cause the redundancy of resources in the eastern region, which is not conducive to the improvement of enterprises’ innovation enthusiasm. However, the relative resources in the western region are not perfect, which is more likely to stimulate enterprises’ development-oriented innovation and exploratory innovation for resources. Whether there are other reasons for this phenomenon can be further explored in future research.

This article also has some limitations. First, in the sample period, due to the availability of data, the article selects 5 years of data, which reduces the accuracy of research results. In the future, it can continue to expand the research scope and track the development trend of its innovation efficiency; Second, the selection of indicators is not rich enough, and the indicator system can be further enriched in the future. Despite these limitations, this paper follows the principles of truthfulness and objectivity in the process of model selection and data processing, which ensures the objectivity and effectiveness of the conclusions. At the same time, in the future research, we can further expand the research target, and further explore a new perspective for the research of the large health industry by combining the era characteristics of the digital transformation of the health industry and the international vision of the human health community.

## 6. Conclusion and recommendations

Based on panel data for 192 Chinese listed health industry companies from 2015 to 2020, this study constructed an evaluation index for innovation efficiency and used the DEA–Malmquist index model to analyse the innovation efficiency of the companies empirically, tested by σ-convergence and β-convergence models. The research results show that: (1) the innovation efficiency of Chinese listed health industry companies shows a time trend of overall increase and a spatial characteristic of gradual decrease from west to east and from south to north, with heterogeneity by industry. Overall, there is still much room for improvement in the resource allocation efficiency of listed companies in the health sector. (2) The average total factor productivity of Chinese listed health companies is 1.072, indicating a relatively stable growth trend. At the same time, the innovation efficiency Malmquist index is largely determined by changes in technological progress. (3) The innovation efficiency of health industry listed companies in the whole country, North China, South China, and Northwest China showed a σ-convergence trend. The listed companies in China as a whole and in the six regions other than the Northwest region showed absolute β-convergence, and in China, North China, Northeast China, East China, and South China, conditional β-convergence.

Based on the above empirical conclusion, this paper puts forward the following policy recommendations: First, create a good development environment for the health industry. China’s health industry is in the initial stage, and the efficiency of enterprise innovation needs to be improved. Therefore, it also needs national guidance and government support, the introduction of health industry support policies, and the optimization of government services. From a dynamic point of view, the efficiency of enterprise innovation is increasing, but the scale efficiency is not yet optimal, and the index of technological progress plays a decisive role. Therefore, we should actively drive enterprise innovation investment, set up a research base for healthy industries, strengthen Industry-University-Research cooperation, promote the industrialization of technological innovation achievements, truly realize the improvement of technological innovation efficiency through technological progress, and at the same time carry out reasonable scale expansion on the basis of effectively ensuring our own innovative research and development activities. Second, narrow the development gap between regions and maintain coordinated development among regions. Judging from the development status of innovation efficiency in various regions, the resource-rich eastern region did not make full use of its development advantages in the process of enterprise development, resulting in the waste and redundancy of resources to a certain extent. On the one hand, the resources in the eastern region can be appropriately transferred to the western region to attract talents and technology through favorable policies, thus maintaining the development trend in the western region. On the other hand, the eastern region should cultivate and support high-quality enterprises with potential, guide enterprises to merge and integrate through the market mechanism, expand the scale, implement the strategy of large enterprises, eliminate backward production capacity, reduce the waste of resources caused by low-level repeated competition, and solve the problem of low scale efficiency. Third, pay attention to the heterogeneity of industries and formulate different development strategies based on the characteristics of different industries. The health industry is huge and complicated, including both the medical industry with medical services as the main body and the pharmaceutical industry with the production and sales of drugs, medical devices and medical consumables as the main body. From the current innovation efficiency, the innovation efficiency of the pharmaceutical industry is higher than that of the medical service industry. In the long run, the aging process in our country is accelerating, and the demand for medical services is greatly increasing. Therefore, it is necessary to speed up the gathering of recuperation services and health management-related industries with medical and health services as the main body, and to form inter-industry linkage and sharing so as to drive the scale expansion. The pharmaceutical industry accounts for half of the health industry, and knowledge and capital requirements are more intensive. Therefore, the government should allocate scientific research funds rationally, increase investment in research and development, set up special funds for some key projects, adjust tax and financing policies, and provide financial support for enterprises.

## Data availability statement

The datasets presented in this study can be found in online repositories. The names of the repository/repositories and accession number(s) can be found in the article/Supplementary material.

## Author contributions

YH, MiH, and MaH: conceptualization, validation, and supervision. YH, MiH, and JL: methodology. YH and JL: software. MiH, SC, and MaH: formal analysis. YH, XW, and MaH: investigation. YH: resources. MiH and JL: data curation. YH and MiH: writing – original draft preparation and project administration. XW, MaH, and JL: writing – review and editing. MaH and SC: visualization. YH and SC: funding acquisition. All authors contributed to the article and approved the submitted version.

## Funding

This research was funded by the National Key R&D Program of China (2021YFF0601005), Major National Social Science Projects (19ZDA085), China Postdoctoral Science Foundation (grant number: 2020M672317) and the National Natural Science Foundation Youth Project (grant number: 71904045).

## Conflict of interest

The authors declare that the research was conducted in the absence of any commercial or financial relationships that could be construed as a potential conflict of interest.

## Publisher’s note

All claims expressed in this article are solely those of the authors and do not necessarily represent those of their affiliated organizations, or those of the publisher, the editors and the reviewers. Any product that may be evaluated in this article, or claim that may be made by its manufacturer, is not guaranteed or endorsed by the publisher.

## References

[ref1] Aigner D. Lovell C. Schmidt P. (1977). Formulation and estimation of stochastic frontier production function models. J. Econ. 6, 21–37. doi: 10.1016/0304-4076(77)90052-5

[ref2] Barro R. J. (1991). Economic growth in a cross section of countries. Q. Econ. 106, 407–443. doi: 10.2307/2937943

[ref3] Barro R. Sala-i-Martin X. (1992). Convergence. J. Political Econ. 100, 223–251. doi: 10.1086/261816

[ref4] Bresciani S. Puertas R. Ferraris A. Santoro G. (2021). Innovation, environmental sustainability and economic development: DEA-bootstrap and multilevel analysis to compare two regions. Technol. Forecast. Soc. Chang. 172:121040. doi: 10.1016/j.techfore.2021.121040

[ref5] Central Committee of the Communist Party of China, State Council. (n.d.). The “Health China 2030” Planning Outline. Available at: http://www.gov.cn/xinwen/2016-10/25/content_5124174.htm (Accessed September, 2022).

[ref6] Chao K. (2020). Empirical test of technological innovation efficiency of equipment manufacturing industry based on SFA. Stat. Decis. 36, 72–75. doi: 10.13546/j.cnki.tjyjc.2020.20.015

[ref7] Charnes A. Cooper W. W. Rhodes E. (1978). Measuring the efficiency of decision making units. Eur. J. Oper. Res. 2, 429–444.

[ref8] Chen C. (2022). An empirical study on innovation efficiency based on three-stage DEA model: evidence from National Industrial Enterprises from 2005 to 2020. Sci. Decis. Making. 9, 111–121.

[ref9] China Statistics Bureau. (2019). Statistical Classification of the Health Industry (NSO Order No. 27). Available at: http://www.stats.gov.cn/tjgz/tzgb/201904/t20190409_1658560.html (Accessed September, 2022).

[ref10] Fan D. C. Gu X. M. (2022). Analysis on the key influencing factors of technological innovation efficiency in high-tech industries: an empirical study based on DEA-Malmquist and BMA. Sci. Res. Manag. 43, 70–78. doi: 10.19571/j.cnki.1000-2995.2022.01.008

[ref11] Furman J. L. Porter M. E. Stern S. (2002). The determinants of national innovative capacity. Res. Policy 31, 899–933. doi: 10.1016/S0048-7333(01)00152-4

[ref12] Gascón F. Lozano J. Ponte B. Fuente D. (2017). Measuring the efficiency of large pharmaceutical companies: an industry analysis. Eur. J. Health Econ. 18, 587–608. doi: 10.1007/s10198-016-0812-3, PMID: 27344446

[ref13] Haschka R. E. Herwartz H. (2020). Innovation efficiency in European high-tech industries: evidence from a Bayesian stochastic frontier approach. Res. Policy 49:104054. doi: 10.1016/j.respol.2020.104054

[ref14] He F. (2004). Measurement of China's technical efficiency: application of stochastic frontier production function. Sci. Res. Manag. 5, 100–103. doi: 10.19571/j.cnki.1000-2995.2004.05.019

[ref15] Hrazdil K. Trottier K. Zhang R. (2013). A comparison of industry classification schemes: a large sample study. Econ. Lett. 118, 77–80. doi: 10.1016/j.econlet.2012.09.022

[ref16] Jungmittag A. (2006). Innovation dynamics in the EU: convergence or divergence? A cross-country panel data analysis. Empir. Econ. 31, 313–331. doi: 10.1007/s00181-005-0018-5

[ref17] Kádárová J. Durkáčová M. Teplická K. Kádár G. (2015). The proposal of an innovative integrated BSC–DEA model. Procedia Econ. Finance. 23, 1503–1508. doi: 10.1016/S2212-5671(15)00375-5

[ref18] Kaitelidou D. Katharaki M. Kalogeropoulou M. Economou C. Liaropoulos L. (2016). The impact of economic crisis to hospital sector and the efficiency of Greek public hospitals. Eur. J. Bus. Soc. Sci. 4, 111–125.

[ref19] Lai H. B. Shi H. (2021). Research on technology system and innovation efficiency of pharmaceutical manufacturing industry. Sci. Res. Manag. 42, 16–24. doi: 10.19571/j.cnki.1000-2995.2021.11.003

[ref20] Lin B. Luan R. (2020). Do government subsidies promote efficiency in technological innovation of China's photovoltaic enterprises? J. Clean. Prod. 254:120108. doi: 10.1016/j.jclepro.2020.120108

[ref21] Liu G. W. Shao Y. F. Liu B. (2021). Research on the evaluation of collaborative innovation capability of China's big health industry chain from the perspective of modular network. Sci. Technol. Progress Policy 38, 85–95.

[ref22] Liu F. C. Zhang N. Zhao L. S. (2020). Research on the evaluation of innovation efficiency of high-tech manufacturing industry in the three northeastern provinces – analysis based on two-stage network DEA model. Manag. Rev. 32, 90–103. doi: 10.14120/j.cnki.cn11-5057/f.2020.04.008

[ref23] Lv Y. W. Xie Y. X. Lou X. J. (2020). Research on space-time transition and convergence trend of regional green innovation efficiency in China. J. Quant. Technol. Econ. 37, 78–97. doi: 10.13653/j.cnki.jqte.2020.05.005

[ref24] Malmquist S. (1953). Index numbers and indifference surfaces. Trab. Estad. 4, 209–242. doi: 10.1007/BF03006863

[ref600] Mankiw G. Romer D. Weil D. N. (1992). A Contribution to the Empirics of Economic Growth. Q. J. Econ. 107, 407–437.

[ref25] Meeusen W. Julien V. D. B. (1977). Efficiency estimation from cobb-Douglas production functions with composed error. Int. Econ. Rev. 18, 435–444. doi: 10.2307/2525757

[ref26] Nasierowski W. Arcelus F. J. (2003). On the efficiency of national innovation systems. Socio Econ. Plan. Sci. 37, 215–234. doi: 10.1016/S0038-0121(02)00046-0

[ref27] Pan W. He Z. Pan H. Peng H. Jian M. (2021). The development of big health industry: a perspective of industrial chain and industrial system construction. Sci. Decis. Mak. 3, 36–61.

[ref28] Patel P. Pavitt K. (1994). Uneven and divergent technological accumulation among advanced countries: Evidenceand a framework of explation. Ind. Corporate Changes. 3, 759–787. doi: 10.1093/icc/3.3.759

[ref29] Ray S. C. Desli E. (1997). Productivity growth, technical progress, and efficiency change in industrialized countries: comment. Am. Econ. Rev. 87, 1033–1039.

[ref30] Shen N. Peng H. Yao J. (2019). A study on the spillover and innovation efficiency of multi-channel international research and development and the spatial convergence. Stud. Sci. 37, 1091–1101+1141. doi: 10.16192/j.cnki.1003-2053.2019.06.015

[ref31] Shin K. Lee D. Shin K. Kim E. (2018). Measuring the efficiency of U.S. pharmaceutical companies based on open innovation types. J. Open Innov. Technol. Mark. Complex. 4:34. doi: 10.3390/joitmc4030034

[ref32] Sisodia A. Jindal R. (2021). A meta-analysis of industry 4.0 design principles applied in the health sector. Eng. Appl. Artif. Intell. 104:104377. doi: 10.1016/j.engappai.2021.104377

[ref33] State Council. (n.d.). Implementing Health China Action. Available at: http://www.gov.cn/zhengce/content/2019-07/15/content_5409492.htm (Accessed September, 2022).

[ref34] Sun J. L. Ma L. Q. (2019). Research on technological innovation efficiency and regional differences of state-owned high-tech manufacturing industry based on DEA-Malmquist index. J. Southeast Univ. (Philos. Soc. Sci) 21, 114–118. doi: 10.13916/j.cnki.issn1671-511x.2019.s1.024

[ref35] Wang R. Q. Peng L. T. (2010). Measurement of regional technological innovation efficiency and analysis of influencing factors based on SFA and Malmquist methods. Sci. Sci. Manage. S.&T. 31, 121–128.

[ref36] Xiao L. M. Gao J. F. Liu S. (2017). The changing trend of regional green technology innovation efficiency in China based on spatial gradient—empirical analysis of provincial panel data. Soft Sci. 31, 63–68. doi: 10.13956/j.ss.1001-8409.2017.09.14

[ref37] Zhang X. Guo S. F. (2022). A study on the convergence of industrial technology innovation efficiency in Chinese provinces from the perspective of spatial effects. Survey World 1, 48–57. doi: 10.13778/j.cnki.11-3705/c.2022.01.006

[ref38] Zhang M. Y. Wen S. H. Han D. H. (2011). DEA analysis on regional innovation efficiency of China. World Survey Res. 6, 31–33. doi: 10.13778/j.cnki.11-3705/c.2011.06.009

[ref39] Zhang T. Yu B. Y. (2019). Research on the evaluation of the efficiency of the innovation system of strategic emerging industries in China–taking the medical device industry as an example. Jiangsu Soc. Sci. 3, 76–85. doi: 10.13858/j.cnki.cn32-1312/c.2019.03.010

[ref40] Zhao Q. Z. Liu Z. X. Cui H. R. (2023). Research on efficiency measurement and spatial convergence of technological innovation of high-tech Industries in China. Stat. Decis. 1, 183–188. doi: 10.13546/j.cnki.tjyjc.2023.01.034

[ref41] Zhong S. Liang S. Q. Zhong Y. X. Zheng Y. Y. Wang F. J. (2022). Measure on innovation efficiency of China's pharmaceutical manufacturing industry. Front. Public Health 10:1024997. doi: 10.3389/FPUBH.2022.1024997, PMID: 36504962 PMC9731224

