# Supplementary material for: Change and convergence of innovation efficiency among listed health companies in China: Empirical study based on the DEA–Malmquist model
Source: Front Psychol. 2023 Mar 9;14:1100717. doi: 10.3389/fpsyg.2023.1100717 (PMC10033529; doi:10.3389/fpsyg.2023.1100717)
Supplement: Supplementary file 1 [file Data_Sheet_1.doc]

**Appendix A**

**Table A1.**

**Innovation efficiency values of 192 listed companies in the health industry 2016-2020.**

| **Company Name** | **Year 2016** | **Year 2017** | **Year 2018** | **Year 2019** | **Year 2020** |
| --- | --- | --- | --- | --- | --- |
| Northeast Pharmaceutical Group Co., Ltd. | 0.318 | 0.45 | 0.481 | 0.441 | 0.459 |
| Jilin Aodong Pharmaceutical Group Co., Ltd. | 0.98 | 1 | 0.764 | 0.887 | 0.76 |
| Changchun High and New Technology Industries (Group) Inc. | 0.362 | 0.379 | 0.538 | 0.725 | 0.666 |
| Tonghua Golden-Horse Pharmaceutical Industey Co. Ltd. | 0.74 | 0.563 | 0.641 | 0.413 | 0.425 |
| Jilin Zixin Pharmaceutical Industrial Co., Ltd. | 0.659 | 0.695 | 0.737 | 0.836 | 0.5 |
| Harbin Gloria Pharmaceuticals Co., Ltd. | 0.584 | 0.621 | 0.701 | 0.492 | 0.665 |
| Jilin Jian Yisheng Pharmaceutical Co., Ltd. | 0.486 | 0.532 | 0.652 | 0.732 | 0.513 |
| Sunflower Pharmaceutical Group Co., Ltd | 0.54 | 0.507 | 0.634 | 0.626 | 0.462 |
| Ji Yao Holding Group Co., Ltd. | 0.687 | 0.682 | 0.76 | 0.565 | 0.461 |
| Dirui Industrial Co., Ltd. | 0.449 | 0.444 | 0.662 | 0.651 | 0.575 |
| Harbin Pharmaceutical Group Co., Ltd. | 0.529 | 0.522 | 0.614 | 0.68 | 0.453 |
| Tonghua Dongbao Pharmaceutical Co., Ltd. | 0.575 | 0.612 | 0.616 | 0.675 | 0.531 |
| HeiLongJiang ZBD Pharmaceutical Co., Ltd. | 0.419 | 0.473 | 0.739 | 0.78 | 0.664 |
| Pacific Shuanglin Bio-pharmacy Co., LTD | 0.758 | 0.755 | 0.73 | 0.876 | 0.662 |
| Beijing Centergate Technologies (Holding) Co., Ltd. | 0.678 | 0.638 | 0.712 | 0.771 | 0.509 |
| Beijing SL Pharmaceutical Co., Ltd. | 0.457 | 0.526 | 0.587 | 0.691 | 0.481 |
| Tianjin Lisheng Pharmaceutical Co., Ltd. | 0.51 | 0.464 | 0.552 | 0.664 | 0.439 |
| Andon Health Co.,Ltd. | 0.413 | 0.354 | 0.428 | 0.5 | 0.459 |
| Shijiazhuang Yiling Pharmaceutical Co., Ltd. | 0.326 | 0.402 | 0.428 | 0.519 | 0.377 |
| Jinhe Biotechnology Co., Ltd. | 0.576 | 0.511 | 0.602 | 0.742 | 0.54 |
| Beijing Aosaikang Pharmaceutical Co., Ltd. | 0.528 | 0.618 | 0.855 | 0.638 | 0.431 |
| Lepu Medical Technology (Beijing) Co., Ltd. | 0.407 | 0.457 | 0.486 | 0.463 | 0.478 |
| Beijing Beilu Pharmaceutical Co., Ltd. | 0.538 | 0.72 | 0.768 | 0.924 | 0.637 |
| Tianjin Chase Sun Pharmaceutical Co., Ltd. | 0.458 | 0.374 | 0.445 | 0.557 | 0.446 |
| Tianjin Ringpu Bio-technology Co., Ltd. | 0.513 | 0.553 | 0.587 | 0.707 | 0.626 |
| Shanxi Zhendong Pharmaceutical Co., Ltd | 0.567 | 0.591 | 0.515 | 0.677 | 0.568 |
| Staidson(Beijing) BioPharmaceuticals Co., Ltd. | 0.554 | 0.513 | 0.594 | 0.656 | 0.417 |
| Baotou Dongbao Bio-Tech Co., Ltd. | 0.642 | 0.643 | 0.726 | 0.857 | 0.616 |
| Shanxi C&Y Pharmaceutical Group Co., Ltd | 0.48 | 0.51 | 0.634 | 0.687 | 0.468 |
| Hebei Changshan Biochemical Pharmaceutical Co., Ltd. | 0.59 | 0.59 | 0.685 | 0.717 | 0.528 |
| Beijing Leadman Biochemistry Co., Ltd. | 0.617 | 0.61 | 0.69 | 0.803 | 0.607 |
| Beijing Strong Biotechnologies,Inc. | 0.747 | 0.664 | 0.705 | 0.805 | 0.63 |
| Beijing Science Sun Pharmaceutical Co., Ltd. | 0.602 | 0.66 | 0.748 | 0.833 | 0.663 |
| Beijing Wandong Medical Technology Co., Ltd. | 0.531 | 0.478 | 0.645 | 0.766 | 0.587 |
| China Meheco Group Co., Ltd. | 0.894 | 1 | 0.817 | 0.725 | 0.791 |
| China Resources Double-Crane Pharmaceutical Co., Ltd. | 0.464 | 0.547 | 0.655 | 0.714 | 0.52 |
| Beijing Tongrentang Co., Ltd. | 0.519 | 0.554 | 0.587 | 0.747 | 0.592 |
| Beijing Tiantan Biological Products Co., Ltd | 0.459 | 0.749 | 0.826 | 0.908 | 0.731 |
| China Animal Husbandry Industry Co., Ltd. | 0.276 | 0.326 | 0.477 | 0.53 | 0.42 |
| Jinyu Bio-technology Co., Ltd. | 0.784 | 0.885 | 0.877 | 0.704 | 0.548 |
| Bluestar Adisseo Company | 1 | 0.923 | 0.803 | 0.911 | 0.812 |
| Tianjin Zhongxin Pharmaceutical Group Co., Ltd | 0.489 | 0.525 | 0.591 | 0.686 | 0.483 |
| Yabao Pharmaceutical Group Co., Ltd. | 0.347 | 0.369 | 0.448 | 0.553 | 0.599 |
| Tianjin Tianyao Pharmaceutical Co., Ltd. | 0.478 | 0.568 | 0.626 | 0.615 | 0.406 |
| Tasly Pharmaceutical Group Co., Ltd. | 0.27 | 0.313 | 0.331 | 0.409 | 0.311 |
| GuangYuYuan Chinese Herbal Medicine Co., Ltd. | 0.823 | 0.723 | 0.711 | 0.667 | 0.442 |
| North China Pharmaceutical Co., Ltd | 0.35 | 0.47 | 0.482 | 0.532 | 0.426 |
| Anhui Fengyuan Pharmaceutical Co., Ltd. | 0.573 | 0.626 | 0.632 | 0.735 | 0.576 |
| Dong-E-E-Jiao Co., Ltd. | 0.781 | 0.853 | 0.785 | 0.643 | 0.431 |
| Renhe Pharmacy Co., Ltd. | 0.793 | 0.697 | 0.783 | 0.825 | 0.612 |
| Apeloa Pharmaceutical Co., Ltd | 0.302 | 0.341 | 0.455 | 0.561 | 0.454 |
| Shandong Wit Dyne Health Co., Ltd | 0.581 | 0.596 | 0.645 | 0.792 | 0.591 |
| Jinling Pharmaceutical Company Limited | 0.584 | 0.597 | 0.723 | 0.833 | 0.84 |
| Zhejiang NHU Co., Ltd. | 0.408 | 0.441 | 0.558 | 0.432 | 0.522 |
| Yifan Pharmaceutical Co., Ltd. | 0.813 | 0.437 | 0.408 | 0.467 | 0.474 |
| Zhejiang Jingxin Pharmaceutical Co., Ltd. | 0.401 | 0.417 | 0.497 | 0.589 | 0.476 |
| Shanghai Kehua Bio-Engineering Co., Ltd. | 0.574 | 0.524 | 0.644 | 0.774 | 0.706 |
| Meinian Onehealth Healthcare Holdings Co., Ltd. | 1 | 1 | 1 | 0.578 | 0.574 |
| Wanbanged Pharmaceutical Holding Group Co., Ltd. | 0.566 | 0.741 | 0.736 | 0.806 | 0.86 |
| Zhejiang Hisoar Pharmaceutical Co., Ltd. | 0.415 | 0.447 | 0.577 | 0.736 | 0.457 |
| Shandong Wohua Pharmaceutical Co., Ltd. | 0.552 | 0.55 | 0.654 | 0.758 | 0.544 |
| Everjoy Health Group Co., Ltd. | 0.498 | 0.492 | 0.624 | 0.705 | 0.604 |
| Jiangsu Aoyang Health Industry Co. ltd. | 0.744 | 0.688 | 0.698 | 0.59 | 0.48 |
| Jiangsu Yuyue Medical Equipment and Supply Co., Ltd. | 0.394 | 0.493 | 0.583 | 0.644 | 0.469 |
| Shanghai RAAS Blood Products Co., Ltd. | 1 | 0.738 | 0.373 | 0.858 | 0.821 |
| Jiangsu Nhwa Pharmaceutical Co., Ltd | 0.511 | 0.525 | 0.595 | 0.695 | 0.565 |
| Zhejiang Xianju Pharmaceutical Co., Ltd. | 0.45 | 0.614 | 0.627 | 0.714 | 0.467 |
| Jinghua Pharmaceutical Group Co., Ltd. | 0.632 | 0.514 | 0.676 | 0.69 | 0.66 |
| Zhejiang Yatai Pharmaceutical Co., Ltd. | 0.534 | 0.557 | 0.627 | 0.367 | 0.501 |
| Blue Sail Medical Co.,Ltd. | 0.665 | 0.555 | 0.66 | 0.613 | 0.563 |
| ChangjiangRunfa Health Industry Co., Ltd. | 0.651 | 0.543 | 0.641 | 0.709 | 0.591 |
| Changzhou Qianhong Biopharma Co., Ltd. | 0.615 | 0.59 | 0.665 | 0.793 | 0.509 |
| Brother Enterprises Holding Co., Ltd. | 0.586 | 0.675 | 0.572 | 0.712 | 0.588 |
| Shandong Sinobioway Biomedicine Co., Ltd. | 0.74 | 0.669 | 0.655 | 0.833 | 0.526 |
| Yantai Dongcheng Biochemicals Co., Ltd | 0.635 | 0.62 | 0.773 | 0.82 | 0.631 |
| Anhui Anke Biotechnology (Group)Co., Ltd. | 0.587 | 0.52 | 0.561 | 0.6 | 0.383 |
| Shanghai Kaibao Pharmaceutical CO., Ltd. | 0.463 | 0.473 | 0.555 | 0.625 | 0.5 |
| Hangzhou Century Co., Ltd. | 0.522 | 0.606 | 0.526 | 0.64 | 0.449 |
| Huaren Pharmaceutical Co., Ltd. | 0.471 | 0.531 | 0.655 | 0.704 | 0.502 |
| Zhejiang Sunflower Great Health Limited Liability Company | 0.471 | 0.512 | 0.447 | 0.868 | 0.587 |
| INKON Life Technology Co., Ltd. | 0.721 | 0.674 | 0.81 | 0.776 | 0.72 |
| Zhejiang Jolly Pharmaceutical Co., Ltd | 0.568 | 0.572 | 0.673 | 0.818 | 0.566 |
| Shandong Jincheng Pharmaceutical Group Co., Ltd | 0.467 | 0.622 | 0.529 | 0.587 | 0.678 |
| Dian Diagnostics Group Co., Ltd. | 0.45 | 0.485 | 0.564 | 0.607 | 0.639 |
| Youngy Health Co., Ltd. | 0.797 | 0.947 | 1 | 0.707 | 0.962 |
| Winning Health Technology Group Co., Ltd. | 0.413 | 0.322 | 0.325 | 0.383 | 0.389 |
| Boya Bio-pharmaceutical Group Co., Ltd | 0.65 | 0.743 | 0.735 | 0.902 | 0.707 |
| Ningbo David Medical Device Co., Ltd. | 0.722 | 0.738 | 0.728 | 0.953 | 0.697 |
| Hangzhou Tigermed Consulting Co., Ltd. | 0.586 | 0.578 | 0.646 | 0.744 | 0.679 |
| Zhejiang Wolwo Bio-Pharmaceutical Co., Ltd. | 0.658 | 0.676 | 0.778 | 0.867 | 0.578 |
| Fujian Cosunter Pharmaceutical Co., Ltd. | 0.612 | 0.641 | 0.698 | 0.755 | 0.637 |
| Medicalsystem Biotechnology Co., Ltd. | 0.61 | 0.686 | 0.923 | 0.638 | 0.744 |
| Anhui Shanhe Pharmaceutical Excipients Co., Ltd. | 0.662 | 0.667 | 0.752 | 0.854 | 0.608 |
| Jiangxi Sanxin Medtec Co., Ltd. | 0.567 | 0.607 | 0.695 | 0.847 | 0.591 |
| Jiangxi Fushine Pharmaceutical Co., Ltd. | 0.639 | 0.6 | 0.671 | 0.786 | 0.536 |
| Shanghai Fosun Pharmaceutical (Group) Co., Ltd. | 0.4 | 0.446 | 0.182 | 0.197 | 0.297 |
| JiangSu WuZhong Pharmaceutical Development Co., Ltd. | 0.643 | 0.596 | 0.57 | 0.752 | 0.486 |
| Zhejiang Medicine Co., Ltd. | 0.292 | 0.269 | 0.354 | 0.395 | 0.324 |
| Zhejiang Hisun Pharmaceutical Co., Ltd. | 0.157 | 0.232 | 0.262 | 0.313 | 0.345 |
| Jiangsu Hengrui Medicine Co., Ltd. | 0.259 | 0.25 | 0.324 | 0.304 | 0.261 |
| Shanghai Shyndec Pharmaceutical Co., Ltd. | 0.572 | 0.495 | 0.43 | 0.446 | 0.398 |
| Zhangzhou Pientzehuang Pharmaceutical Co., Ltd. | 0.693 | 0.824 | 0.881 | 1 | 0.894 |
| Jiangsu Lianhuan Pharmaceutical Co., Ltd. | 0.626 | 0.654 | 0.691 | 0.857 | 0.602 |
| Zhe Jiang Huahai Pharmaceutical Co., Ltd. | 0.287 | 0.255 | 0.262 | 0.33 | 0.298 |
| Shanghai Jiaoda Onlly Co., Ltd. | 0.682 | 0.701 | 0.631 | 0.849 | 0.656 |
| Jiangsu Kanion Pharmaceutical Co., Ltd. | 0.609 | 0.546 | 0.611 | 0.768 | 0.609 |
| Zhejiang Conba Pharmaceutical Co., Ltd. | 0.429 | 0.509 | 0.605 | 0.473 | 0.457 |
| Shinva Medical Instrument Co., Ltd. | 0.784 | 0.887 | 0.7 | 1 | 1 |
| Shanghai Shenqi Pharmaceutical Investment Management Co., Ltd. | 0.751 | 0.86 | 0.781 | 0.86 | 0.632 |
| Shanghai Fudan Forward S&T Co., Ltd | 0.471 | 0.509 | 0.628 | 0.723 | 0.487 |
| NanJing Pharmaceutical Company Limited | 1 | 1 | 1 | 1 | 1 |
| Jiangzhong Pharmaceutical Co., Ltd. | 0.596 | 0.616 | 0.722 | 0.855 | 0.876 |
| Ningbo Sanxing Medical Electric Co., Ltd. | 0.487 | 0.952 | 0.846 | 1 | 0.842 |
| Shanghai Pharmaceuticals Holding Co., Ltd | 0.758 | 1 | 1 | 1 | 1 |
| Zhejiang Shapuaisi Pharmaceutical Co., Ltd. | 0.64 | 0.651 | 0.635 | 0.805 | 0.543 |
| Chimin Health Management Co., Ltd. | 0.587 | 0.603 | 0.719 | 0.841 | 0.747 |
| Zhejiang Jiuzhou Pharmaceutical Co., Ltd. | 0.412 | 0.454 | 0.57 | 0.664 | 0.45 |
| Shanghai Hile Bio-Technology Co., Ltd. | 0.644 | 0.741 | 0.665 | 0.802 | 0.624 |
| China Reform Health Management and Services Group Co., Ltd. | 0.347 | 0.33 | 0.418 | 0.602 | 0.472 |
| Livzon Pharmaceutical Group Inc. | 0.439 | 1 | 0.523 | 0.613 | 0.665 |
| Hainan Haiyao Co., Ltd. | 0.638 | 0.652 | 0.708 | 0.784 | 0.747 |
| China Resources Sanjiu Medical & Pharmaceutical Co., Ltd. | 0.962 | 0.887 | 0.768 | 1 | 0.956 |
| Daan Gene Co., Ltd. | 0.526 | 0.696 | 0.672 | 0.855 | 1 |
| HL Corp (Shenzhen) | 0.448 | 0.488 | 0.582 | 0.626 | 0.478 |
| Guilin Layn Natural Ingredients Corp. | 0.655 | 0.74 | 0.779 | 0.929 | 0.668 |
| Guangdong Jiaying Pharmaceutical Co., Ltd. | 0.625 | 0.551 | 0.731 | 0.815 | 0.607 |
| Guilin Sanjin Pharmaceutical Co., Ltd. | 0.583 | 0.631 | 0.685 | 0.793 | 0.616 |
| Shenzhen Salubris Pharmaceutical Co., Ltd. | 0.406 | 0.429 | 0.488 | 0.456 | 0.403 |
| Guangdong Zhongsheng Pharmaceutical Co., Ltd. | 0.462 | 0.423 | 0.545 | 0.613 | 0.39 |
| Shenzhen Hepalink Pharmaceutical Group Co., Ltd. | 0.543 | 0.579 | 0.732 | 0.825 | 0.583 |
| Guangdong Taiantang Pharmaceutical Co., Ltd. | 0.736 | 0.766 | 0.761 | 0.869 | 0.631 |
| Shenzhen Glory Medical Co., Ltd. | 0.498 | 0.607 | 0.718 | 0.849 | 0.574 |
| Hainan Shuangcheng Pharmaceuticals Co., Ltd. | 0.503 | 0.61 | 0.685 | 0.86 | 0.597 |
| Teyi Pharmaceuticals Group Co., Ltd | 0.573 | 0.56 | 0.657 | 0.79 | 0.536 |
| Improve Medical Instruments Co., Ltd | 0.466 | 0.558 | 0.637 | 0.76 | 0.616 |
| Honz Pharmaceutical Co., Ltd. | 0.558 | 0.604 | 0.713 | 0.595 | 0.512 |
| By-health Co., Ltd | 0.636 | 0.659 | 0.849 | 0.722 | 0.907 |
| Xiangxue Pharmaceutical Co., Ltd. | 0.358 | 0.413 | 0.517 | 0.564 | 0.63 |
| ChemPartner PharmaTech Co., Ltd. | 0.632 | 0.758 | 0.794 | 0.61 | 0.53 |
| Hybio Pharmaceutical Co., Ltd. | 0.555 | 0.578 | 0.531 | 0.488 | 0.392 |
| Zhuhai Hokai Medical Instruments Co., Ltd. | 0.505 | 0.475 | 0.581 | 0.705 | 0.567 |
| Guangdong Vtr Bio-Tech Co., Ltd. | 0.509 | 0.421 | 0.615 | 0.741 | 0.585 |
| Boji Medical Technology Co., Ltd. | 0.645 | 0.644 | 0.725 | 0.821 | 0.631 |
| Guangzhou Wondfo Biotech Co., Ltd | 0.573 | 0.559 | 0.626 | 0.675 | 0.656 |
| Guangxi Wuzhou Zhongheng Group Co., Ltd. | 0.941 | 0.855 | 0.899 | 1 | 0.621 |
| Joincare Pharmaceutical Group Industry Co., Ltd. | 0.302 | 0.711 | 0.363 | 0.475 | 0.386 |
| Well Lead Medical Co., Ltd | 0.587 | 0.662 | 0.762 | 0.74 | 0.514 |
| Tus- Pharmaceutical Group Co., Ltd. | 0.672 | 0.65 | 0.745 | 0.898 | 0.628 |
| Hunan Jingfeng Pharmaceutical Co., Ltd. | 0.561 | 0.388 | 0.48 | 0.427 | 0.302 |
| Hubei Guangji Pharmaceutical Co., Ltd. | 0.628 | 0.624 | 0.702 | 0.743 | 0.522 |
| Jiuzhitang Co., Ltd. | 0.905 | 0.603 | 0.582 | 0.698 | 0.491 |
| Hualan Biological Engineering,Inc. | 0.644 | 0.57 | 0.642 | 0.698 | 0.586 |
| Qianjiang Yongan Pharmaceutical Co., Ltd. | 0.5 | 0.623 | 0.701 | 0.838 | 0.628 |
| Hunan Hansen Pharmaceutical Co., Ltd. | 0.511 | 0.518 | 0.662 | 0.756 | 0.858 |
| Aier Eye Hospital Group Co., Ltd. | 0.863 | 0.937 | 1 | 0.91 | 0.822 |
| Hunan Er-Kang Pharmaceutical Co., Ltd. | 0.763 | 0.533 | 0.656 | 0.788 | 0.599 |
| Humanwell Healthcare (Group) Co., Ltd | 0.359 | 0.42 | 0.285 | 0.422 | 0.408 |
| Henan Taloph Pharmaceutical Stock Co., Ltd. | 0.562 | 0.539 | 0.604 | 0.664 | 0.458 |
| Henan Lingrui Pharmaceutical Co., Ltd | 0.565 | 0.552 | 0.683 | 0.762 | 0.537 |
| Hubei Jumpcan Pharmaceutical Co., Ltd | 0.555 | 0.599 | 0.691 | 0.721 | 0.486 |
| Mayinglong Pharmaceutical Group Co., Ltd. | 0.597 | 0.639 | 0.7 | 0.802 | 0.725 |
| Pulike Biological Engineering,Inc. | 0.601 | 0.618 | 0.672 | 0.734 | 0.491 |
| Yifeng Pharmacy Chain Co., Ltd. | 0.802 | 0.713 | 0.777 | 0.83 | 0.678 |
| Hunan Fangsheng Pharmaceutical Co., Ltd. | 0.517 | 0.522 | 0.688 | 0.879 | 0.548 |
| Dezhan Healthcare Company Limited | 1 | 0.816 | 0.808 | 0.843 | 0.688 |
| YanAn Bicon Pharmaceutical Listed Company | 1 | 1 | 0.519 | 0.601 | 0.461 |
| LanZhou Foci Pharmaceutical Co., Ltd. | 0.624 | 0.608 | 0.699 | 0.794 | 0.57 |
| Ginwa Enterprise (Group) Inc. | 0.853 | 0.875 | 0.764 | 0.895 | 0.632 |
| Yunnan Baiyao Group Co., Ltd | 1 | 1 | 1 | 1 | 1 |
| PKU HealthCare Co., Ltd. | 0.625 | 0.669 | 0.725 | 0.836 | 0.587 |
| Chengdu huasun technology group Inc., Ltd. | 0.552 | 0.586 | 0.67 | 0.797 | 0.623 |
| Huapont Life Sciences Co., Ltd. | 0.356 | 0.473 | 0.479 | 0.501 | 0.483 |
| Tibet Cheezheng Tibetan Medicine Co., Ltd. | 0.571 | 0.593 | 0.687 | 0.785 | 0.554 |
| Guizhou Xinbang Pharmaceutical Co., Ltd. | 1 | 0.796 | 0.537 | 0.871 | 0.597 |
| Sichuan Kelun Pharmaceutical Co., Ltd. | 0.303 | 0.344 | 0.286 | 0.329 | 0.241 |
| Guizhou Bailing Group Pharmaceutical Co., Ltd | 0.711 | 0.583 | 0.664 | 0.777 | 0.627 |
| Haisco Pharmaceutical Group Co., Ltd | 0.351 | 0.422 | 0.515 | 0.598 | 0.373 |
| Kunming Longjin Pharmaceutical Co., Ltd. | 0.703 | 0.713 | 0.755 | 0.861 | 0.696 |
| Chengdu Kanghong Pharmaceutical Group Co., Ltd | 0.477 | 0.466 | 0.52 | 0.586 | 0.302 |
| Chongqing Lummy Pharmaceutical Co., Ltd | 0.507 | 0.649 | 0.683 | 0.744 | 0.521 |
| Chongqing Zhifei Biological Products Co., Ltd. | 0.433 | 0.542 | 0.772 | 0.991 | 0.959 |
| Walvax Biotechnology Co., Ltd. | 0.348 | 0.398 | 0.739 | 0.756 | 0.788 |
| Fuan Pharmaceutical (Group) Co., Ltd. | 0.499 | 0.477 | 0.493 | 0.659 | 0.643 |
| Porton Pharma Solutions Ltd. | 0.52 | 0.526 | 0.568 | 0.684 | 0.452 |
| Sichuan Goldstone Asia Pharmaceutical Inc. | 0.697 | 0.75 | 0.7 | 0.77 | 0.508 |
| Maccura Biotechnology Co., Ltd | 0.618 | 0.583 | 0.78 | 0.784 | 0.571 |
| Chongqing Taiji Industry (Group) Co., Ltd. | 0.794 | 0.593 | 0.62 | 0.589 | 0.469 |
| Tibet Rhodiola Pharmaceutical Holding Company | 0.828 | 0.814 | 0.833 | 0.978 | 0.754 |
| Guizhou Salvage Pharmaceutical Co., Ltd | 0.512 | 0.622 | 0.722 | 0.521 | 0.55 |
| KPC Pharmaceuticals, Inc. | 0.649 | 0.728 | 0.731 | 0.827 | 0.618 |
| Guizhou Yibai Pharmaceutical Co., Ltd. | 0.766 | 0.779 | 0.642 | 0.928 | 0.709 |
| Lionco Pharmaceutical Group Co., Ltd. | 0.521 | 0.573 | 0.631 | 0.771 | 0.54 |

**Appendix B**

**Table A2.** Innovation efficiency MI index and decomposition volume of 192 listed companies in health industry

| **Company Name** | **Technical Efficiency** | **Technological Advances** | **Pure Technology Efficiency** | **Scale Efficiency** | **Malmquist Index** |
| --- | --- | --- | --- | --- | --- |
| Northeast Pharmaceutical Group Co., Ltd. | 1.096 | 1.056 | 1.043 | 1.051 | 1.158 |
| Jilin Aodong Pharmaceutical Group Co., Ltd. | 0.939 | 1.07 | 0.94 | 0.999 | 1.004 |
| Changchun High and New Technology Industries (Group) Inc. | 1.165 | 1.061 | 1.135 | 1.026 | 1.236 |
| Tonghua Golden-Horse Pharmaceutical Industey Co., Ltd. | 0.871 | 1.084 | 0.87 | 1.001 | 0.943 |
| Jilin Zixin Pharmaceutical Industrial Co., Ltd. | 0.933 | 1.048 | 0.937 | 0.996 | 0.978 |
| Harbin Gloria Pharmaceuticals Co., Ltd. | 1.033 | 1.03 | 1.025 | 1.008 | 1.064 |
| Jilin Jian Yisheng Pharmaceutical Co., Ltd. | 1.014 | 1.07 | 1.097 | 0.924 | 1.085 |
| Sunflower Pharmaceutical Group Co., Ltd | 0.962 | 1.1 | 0.968 | 0.993 | 1.058 |
| Ji Yao Holding Group Co., Ltd. | 0.905 | 1.056 | 0.902 | 1.003 | 0.956 |
| Dirui Industrial Co., Ltd. | 1.064 | 1.078 | 1.065 | 0.999 | 1.147 |
| Harbin Pharmaceutical Group Co., Ltd. | 0.962 | 1.051 | 0.935 | 1.028 | 1.01 |
| Tonghua Dongbao Pharmaceutical Co., Ltd. | 0.98 | 1.089 | 0.967 | 1.013 | 1.068 |
| HeiLongJiang ZBD Pharmaceutical Co., Ltd. | 1.122 | 1.039 | 1.119 | 1.003 | 1.165 |
| Pacific Shuanglin Bio-pharmacy Co., Ltd | 0.967 | 1.041 | 1.013 | 0.954 | 1.007 |
| Beijing Centergate Technologies (Holding) Co., Ltd. | 0.931 | 1.068 | 0.932 | 0.999 | 0.995 |
| Beijing SL Pharmaceutical Co., Ltd. | 1.013 | 1.079 | 0.992 | 1.022 | 1.093 |
| Tianjin Lisheng Pharmaceutical Co., Ltd. | 0.963 | 1.074 | 0.964 | 0.999 | 1.035 |
| Andon Health Co., Ltd. | 1.027 | 1.073 | 1.025 | 1.002 | 1.102 |
| Shijiazhuang Yiling Pharmaceutical Co., Ltd. | 1.037 | 1.101 | 1.056 | 0.983 | 1.142 |
| Jinhe Biotechnology Co., Ltd. | 0.984 | 1.07 | 0.995 | 0.989 | 1.053 |
| Beijing Aosaikang Pharmaceutical Co., Ltd. | 0.95 | 1.058 | 0.97 | 0.979 | 1.005 |
| Lepu Medical Technology (Beijing) Co., Ltd. | 1.041 | 1.095 | 1.094 | 0.952 | 1.139 |
| Beijing Beilu Pharmaceutical Co., Ltd. | 1.043 | 1.062 | 1.064 | 0.981 | 1.107 |
| Tianjin Chase Sun Pharmaceutical Co., Ltd. | 0.993 | 1.099 | 0.984 | 1.01 | 1.092 |
| Tianjin Ringpu Bio-technology Co., Ltd. | 1.051 | 1.086 | 1.038 | 1.012 | 1.141 |
| Shanxi Zhendong Pharmaceutical Co., Ltd | 1 | 1.039 | 0.985 | 1.016 | 1.039 |
| Staidson(Beijing) BioPharmaceuticals Co., Ltd. | 0.931 | 1.062 | 0.914 | 1.018 | 0.989 |
| Baotou Dongbao Bio-Tech Co., Ltd. | 0.99 | 1.066 | 1.064 | 0.93 | 1.055 |
| Shanxi C&Y Pharmaceutical Group Co., Ltd | 0.994 | 1.067 | 1.018 | 0.976 | 1.06 |
| Hebei Changshan Biochemical Pharmaceutical Co., Ltd. | 0.973 | 1.044 | 0.963 | 1.01 | 1.016 |
| Beijing Leadman Biochemistry Co., Ltd. | 0.996 | 1.06 | 1.023 | 0.973 | 1.055 |
| Beijing Strong Biotechnologies,Inc. | 0.958 | 1.063 | 0.979 | 0.979 | 1.019 |
| Beijing Science Sun Pharmaceutical Co., Ltd. | 1.024 | 1.062 | 1.033 | 0.991 | 1.088 |
| Beijing Wandong Medical Technology Co., Ltd. | 1.025 | 1.065 | 1.017 | 1.008 | 1.091 |
| China Meheco Group Co., Ltd. | 0.97 | 1.094 | 0.959 | 1.012 | 1.061 |
| China Resources Double-Crane Pharmaceutical Co., Ltd. | 1.029 | 1.07 | 1.004 | 1.025 | 1.101 |
| Beijing Tongrentang Co., Ltd. | 1.033 | 1.088 | 0.997 | 1.037 | 1.125 |
| Beijing Tiantan Biological Products Co., Ltd. | 1.123 | 1.064 | 1.094 | 1.026 | 1.195 |
| China Animal Husbandry Industry Co., Ltd. | 1.111 | 1.08 | 1.034 | 1.074 | 1.2 |
| Jinyu Bio-technology Co., Ltd. | 0.914 | 1.039 | 0.912 | 1.002 | 0.95 |
| Bluestar Adisseo Company | 0.949 | 0.988 | 0.954 | 0.995 | 0.938 |
| Tianjin Zhongxin Pharmaceutical Group Co., Ltd. | 0.997 | 1.11 | 0.972 | 1.026 | 1.106 |
| Yabao Pharmaceutical Group Co., Ltd. | 1.146 | 1.079 | 1.113 | 1.03 | 1.237 |
| Tianjin Tianyao Pharmaceutical Co., Ltd. | 0.96 | 1.076 | 0.956 | 1.005 | 1.033 |
| Tasly Pharmaceutical Group Co., Ltd. | 1.036 | 1.066 | 0.971 | 1.067 | 1.105 |
| GuangYuYuan Chinese Herbal Medicine Co., Ltd. | 0.856 | 1.095 | 0.858 | 0.997 | 0.937 |
| North China Pharmaceutical Co. Ltd. | 1.05 | 1.055 | 1.016 | 1.033 | 1.108 |
| Anhui Fengyuan Pharmaceutical Co., Ltd. | 1.001 | 1.058 | 0.995 | 1.007 | 1.059 |
| Dong-E-E-Jiao Co., Ltd. | 0.862 | 1.059 | 0.84 | 1.026 | 0.913 |
| Renhe Pharmacy Co., Ltd. | 0.937 | 1.073 | 0.991 | 0.946 | 1.006 |
| Apeloa Pharmaceutical Co., Ltd. | 1.107 | 1.048 | 1.058 | 1.046 | 1.16 |
| Shandong Wit Dyne Health Co., Ltd. | 1.004 | 1.09 | 0.992 | 1.012 | 1.095 |
| Jinling Pharmaceutical Co., Ltd. | 1.095 | 1.058 | 1.101 | 0.995 | 1.158 |
| Zhejiang NHU Co., Ltd. | 1.063 | 1.137 | 1.079 | 0.985 | 1.209 |
| Yifan Pharmaceutical Co., Ltd. | 0.874 | 1.066 | 0.903 | 0.967 | 0.932 |
| Zhejiang Jingxin Pharmaceutical Co., Ltd. | 1.044 | 1.052 | 1.037 | 1.006 | 1.098 |
| Shanghai Kehua Bio-Engineering Co., Ltd. | 1.053 | 1.079 | 1.038 | 1.014 | 1.137 |
| Meinian Onehealth Healthcare Holdings Co., Ltd. | 0.87 | 1.092 | 0.939 | 0.927 | 0.95 |
| Wanbangde Pharmaceutical Holding Group Co., Ltd. | 1.11 | 1.023 | 1.097 | 1.012 | 1.136 |
| Zhejiang Hisoar Pharmaceutical Co., Ltd. | 1.024 | 1.094 | 0.988 | 1.036 | 1.12 |
| Shandong Wohua Pharmaceutical Co., Ltd. | 0.996 | 1.071 | 1.033 | 0.965 | 1.067 |
| EVERJOY HEALTH GROUP Co., Ltd. | 1.05 | 1.061 | 1.1 | 0.954 | 1.114 |
| Jiangsu Aoyang Health Industry Co. Ltd. | 0.896 | 1.083 | 0.923 | 0.971 | 0.971 |
| Jiangsu Yuyue Medical Equipment and Supply Co., Ltd. | 1.045 | 1.106 | 1.025 | 1.019 | 1.156 |
| Shanghai RAAS Blood Products Co., Ltd. | 0.952 | 1.055 | 0.965 | 0.986 | 1.004 |
| Jiangsu Nhwa Pharmaceutical Co., Ltd | 1.026 | 1.063 | 1.005 | 1.021 | 1.091 |
| Zhejiang Xianju Pharmaceutical Co., Ltd. | 1.009 | 1.069 | 0.987 | 1.023 | 1.079 |
| Jinghua Pharmaceutical Group Co., Ltd. | 1.011 | 1.074 | 1.046 | 0.966 | 1.085 |
| Zhejiang Yatai Pharmaceutical Co., Ltd. | 0.984 | 1.065 | 0.979 | 1.005 | 1.048 |
| Blue Sail Medical Co., Ltd. | 0.959 | 1.065 | 0.969 | 0.99 | 1.022 |
| ChangjiangRunfa Health Industry Co., Ltd. | 0.976 | 1.071 | 0.969 | 1.007 | 1.045 |
| Changzhou Qianhong Biopharma Co., Ltd. | 0.954 | 1.06 | 0.955 | 0.998 | 1.011 |
| Brother Enterprises Holding Co., Ltd. | 1.001 | 1.089 | 1.019 | 0.983 | 1.09 |
| Shandong Sinobioway Biomedicine Co., Ltd. | 0.918 | 1.068 | 0.933 | 0.984 | 0.981 |
| Yantai Dongcheng Biochemicals Co., Ltd. | 0.998 | 1.074 | 0.995 | 1.003 | 1.072 |
| Anhui Anke Biotechnology (Group)Co., Ltd. | 0.899 | 1.074 | 0.891 | 1.009 | 0.966 |
| Shanghai Kaibao Pharmaceutical Co., Ltd. | 1.019 | 1.102 | 1.101 | 0.925 | 1.123 |
| Hangzhou Century Co., Ltd. | 0.963 | 1.089 | 0.964 | 0.999 | 1.049 |
| Huaren Pharmaceutical Co., Ltd. | 1.016 | 1.057 | 1.04 | 0.977 | 1.074 |
| Zhejiang Sunflower Great Health Limited Liability Company | 1.057 | 1.065 | 1.122 | 0.942 | 1.125 |
| INKON Life Technology Co., Ltd. | 1 | 1.064 | 1.058 | 0.945 | 1.064 |
| Zhejiang Jolly Pharmaceutical Co., Ltd | 0.999 | 1.075 | 1.076 | 0.929 | 1.074 |
| Shandong Jincheng Pharmaceutical Group Co., Ltd | 1.097 | 1.034 | 1.087 | 1.009 | 1.134 |
| Dian Diagnostics Group Co., Ltd. | 1.091 | 1.098 | 1.085 | 1.006 | 1.198 |
| Youngy Health Co., Ltd. | 1.048 | 1.071 | 1.057 | 0.992 | 1.122 |
| Winning Health Technology Group Co., Ltd. | 0.985 | 1.132 | 1.046 | 0.942 | 1.115 |
| Boya Bio-pharmaceutical Group Co., Ltd | 1.021 | 1.077 | 1.016 | 1.006 | 1.1 |
| Ningbo David Medical Device Co., Ltd. | 0.992 | 1.078 | 1.037 | 0.956 | 1.069 |
| Hangzhou Tigermed Consulting Co., Ltd. | 1.038 | 1.095 | 1.046 | 0.992 | 1.136 |
| Zhejiang Wolwo Bio-Pharmaceutical Co., Ltd. | 0.968 | 1.063 | 0.984 | 0.983 | 1.029 |
| Fujian Cosunter Pharmaceutical Co., Ltd. | 1.01 | 1.051 | 1.037 | 0.974 | 1.062 |
| Medicalsystem Biotechnology Co., Ltd. | 1.051 | 1.077 | 1.044 | 1.007 | 1.132 |
| Anhui Shanhe Pharmaceutical Excipients Co., Ltd. | 0.979 | 1.077 | 1.079 | 0.907 | 1.054 |
| Jiangxi Sanxin Medtec Co., Ltd. | 1.01 | 1.086 | 1.089 | 0.928 | 1.097 |
| Jiangxi Fushine Pharmaceutical Co., Ltd. | 0.957 | 1.078 | 1.001 | 0.956 | 1.031 |
| Shanghai Fosun Pharmaceutical (Group) Co., Ltd. | 0.929 | 1.034 | 0.963 | 0.964 | 0.96 |
| JiangSu WuZhong Pharmaceutical Development Co., Ltd. | 0.932 | 1.064 | 0.948 | 0.983 | 0.992 |
| Zhejiang Medicine Co., Ltd. | 1.026 | 1.087 | 1.006 | 1.021 | 1.115 |
| Zhejiang Hisun Pharmaceutical Co., Ltd. | 1.217 | 1.056 | 1.106 | 1.101 | 1.286 |
| Jiangsu Hengrui Medicine Co., Ltd. | 1.002 | 1.045 | 1.076 | 0.931 | 1.047 |
| Shanghai Shyndec Pharmaceutical Co., Ltd. | 0.913 | 1.081 | 0.897 | 1.018 | 0.987 |
| Zhangzhou Pientzehuang Pharmaceutical Co., Ltd. | 1.066 | 1.069 | 1.053 | 1.012 | 1.139 |
| Jiangsu Lianhuan Pharmaceutical Co., Ltd. | 0.99 | 1.074 | 1.02 | 0.971 | 1.063 |
| Zhejiang Ghua Hai Pharmaceutical Co., Ltd. | 1.01 | 1.096 | 1.008 | 1.002 | 1.106 |
| Shanghai Jiaoda Onlly Co., Ltd. | 0.991 | 1.062 | 1.058 | 0.936 | 1.052 |
| Jiangsu Kanion Pharmaceutical Co., Ltd. | 1 | 1.072 | 0.986 | 1.014 | 1.072 |
| Zhejiang Conba Pharmaceutical Co., Ltd. | 1.016 | 1.093 | 0.986 | 1.03 | 1.11 |
| Shinva Medical Instrument Co., Ltd. | 1.063 | 1.152 | 1.054 | 1.009 | 1.224 |
| Shanghai Shenqi Pharmaceutical Investment Management Co., Ltd. | 0.958 | 1.059 | 1.048 | 0.914 | 1.015 |
| Shanghai Fudan Forward S&T Co., Ltd. | 1.008 | 1.078 | 1.04 | 0.97 | 1.087 |
| NanJing Pharmaceutical Co., Ltd | 1 | 1.027 | 1 | 1 | 1.027 |
| Jiangzhong Pharmaceutical Co., Ltd. | 1.101 | 1.074 | 1.122 | 0.981 | 1.183 |
| Ningbo Sanxing Medical Electric Co., Ltd. | 1.147 | 1.094 | 1.11 | 1.033 | 1.255 |
| Shanghai Pharmaceuticals Holding Co., Ltd. | 1.072 | 1.044 | 1 | 1.072 | 1.118 |
| Zhejiang Shapuaisi Pharmaceutical Co., Ltd. | 0.96 | 1.069 | 1.009 | 0.951 | 1.026 |
| Chimin Health Management Co., Ltd. | 1.062 | 1.063 | 1.137 | 0.934 | 1.128 |
| Zhejiang Jiuzhou Pharmaceutical Co., Ltd. | 1.022 | 1.084 | 1.03 | 0.993 | 1.108 |
| Shanghai Hile Bio-Technology Co., Ltd. | 0.992 | 1.067 | 1.046 | 0.949 | 1.059 |
| China Reform Health Management and Services Group Co., Ltd. | 1.08 | 1.108 | 1.176 | 0.919 | 1.197 |
| Livzon Pharmaceutical Group Inc. | 1.11 | 1.036 | 1.105 | 1.004 | 1.15 |
| Hainan Haiyao Co., Ltd. | 1.04 | 1.042 | 1.04 | 1 | 1.084 |
| China Resources Sanjiu Medical & Pharmaceutical Co., Ltd. | 0.998 | 1.062 | 0.989 | 1.009 | 1.06 |
| Daan Gene Co., Ltd. | 1.174 | 1.083 | 1.144 | 1.026 | 1.272 |
| HL Corp (Shenzhen) | 1.017 | 1.09 | 1.099 | 0.925 | 1.108 |
| Guilin Layn Natural Inc. | 1.005 | 1.07 | 1.09 | 0.922 | 1.076 |
| Guangdong Jiaying Pharmaceutical Co., Ltd. | 0.993 | 1.061 | 1.087 | 0.913 | 1.054 |
| Guilin Sanjin Pharmaceutical Co., Ltd. | 1.014 | 1.075 | 1.002 | 1.012 | 1.09 |
| Shenzhen Salubris Pharmaceuticals Co., Ltd. | 0.998 | 1.053 | 0.927 | 1.077 | 1.051 |
| Guangdong Zhongsheng Pharmaceutical Co., Ltd. | 0.959 | 1.087 | 0.933 | 1.028 | 1.042 |
| Shenzhen Hepalink Pharmaceutical Group Co., Ltd. | 1.018 | 1.044 | 1.006 | 1.012 | 1.063 |
| Guangdong Taiantang Pharmaceutical Co., Ltd. | 0.962 | 1.058 | 1.008 | 0.954 | 1.018 |
| Shenzhen Glory Medical Co., Ltd. | 1.036 | 1.077 | 1.088 | 0.952 | 1.115 |
| Hainan Shuangcheng Pharmaceuticals Co., Ltd. | 1.044 | 1.054 | 1.079 | 0.967 | 1.1 |
| Teyi Pharmaceutical Group Co.,Ltd. | 0.984 | 1.075 | 0.992 | 0.992 | 1.057 |
| Improve Medical Instruments Co., Ltd | 1.072 | 1.078 | 1.128 | 0.951 | 1.156 |
| Honz Pharmaceutical Co., Ltd. | 0.979 | 1.116 | 1.102 | 0.888 | 1.092 |
| By-health Co., Ltd. | 1.093 | 1.075 | 1.081 | 1.011 | 1.175 |
| Xiangxue Pharmaceutical Co., Ltd. | 1.152 | 1.084 | 1.129 | 1.02 | 1.249 |
| Chem Partner Pharma Tech Co., Ltd. | 0.957 | 1.125 | 1.02 | 0.938 | 1.077 |
| Hybio Pharmaceutical Co., Ltd. | 0.917 | 1.058 | 0.906 | 1.012 | 0.969 |
| Zhuhai Hokai Medical Instruments Co., Ltd. | 1.029 | 1.07 | 1.046 | 0.984 | 1.102 |
| Guangdong Vtr Bio-Tech Co., Ltd. | 1.035 | 1.081 | 1.039 | 0.996 | 1.119 |
| Boji Medical Technology Co., Ltd. | 0.995 | 1.07 | 1.086 | 0.916 | 1.065 |
| Guangzhou Wondfo Biotech Co., Ltd. | 1.035 | 1.083 | 1.025 | 1.009 | 1.12 |
| Guangxi Wuzhou Zhongheng Group Co., Ltd. | 0.901 | 1.045 | 0.919 | 0.981 | 0.942 |
| Joincare Pharmaceutical Group Industry Co., Ltd. | 1.064 | 1.052 | 1.069 | 0.996 | 1.12 |
| Well Lead Medical Co., Ltd. | 0.967 | 1.092 | 1.043 | 0.927 | 1.057 |
| Tus- Pharmaceutical Group Co., Ltd. | 0.983 | 1.067 | 1.089 | 0.903 | 1.049 |
| Hunan Jingfeng Pharmaceutical Co., Ltd. | 0.857 | 1.087 | 0.85 | 1.007 | 0.931 |
| Hubei Guangji Pharmaceutical Co., Ltd. | 0.955 | 1.068 | 0.988 | 0.967 | 1.02 |
| Jiuzhitang Co., Ltd. | 0.858 | 1.085 | 0.854 | 1.004 | 0.931 |
| Hualan Biological Engineering. Inc. | 0.977 | 1.105 | 0.976 | 1.001 | 1.08 |
| Qianjiang Yongan Pharmaceutical Co., Ltd. | 1.059 | 1.074 | 1.081 | 0.979 | 1.137 |
| Hunan Hansen Pharmaceutical Co., Ltd. | 1.139 | 1.06 | 1.182 | 0.963 | 1.207 |
| Aier Eye Hospital Group Co., Ltd. | 0.988 | 1.059 | 0.987 | 1.001 | 1.046 |
| Hunan Er-Kang Pharmaceutical Co., Ltd. | 0.941 | 1.061 | 0.958 | 0.982 | 0.999 |
| Humanwell Healthcare (Group) Co., Ltd | 1.032 | 1.077 | 1.041 | 0.992 | 1.112 |
| Henan Taloph Pharmaceutical Stock Co., Ltd. | 0.95 | 1.097 | 1.003 | 0.948 | 1.042 |
| Henan Lingrui Pharmaceutical Co., Ltd. | 0.987 | 1.092 | 1.012 | 0.975 | 1.078 |
| Hubei Jumpcan Pharmaceutical Co., Ltd. | 0.967 | 1.089 | 0.947 | 1.021 | 1.053 |
| Mayinglong Pharmaceutical Group Co., Ltd. | 1.05 | 1.074 | 1.075 | 0.977 | 1.128 |
| Pulike Biological Engineering. Inc. | 0.95 | 1.082 | 0.952 | 0.999 | 1.028 |
| Yifeng Pharmacy Chain Co., Ltd. | 0.959 | 1.09 | 1.011 | 0.948 | 1.046 |
| Hunan Fangsheng Pharmaceutical Co., Ltd. | 1.015 | 1.064 | 1.071 | 0.947 | 1.079 |
| Dezhan Healthcare Co., Ltd | 0.911 | 1.071 | 0.915 | 0.995 | 0.975 |
| YanAn Bicon Pharmaceutical Listed Company. | 0.824 | 1.075 | 0.827 | 0.996 | 0.886 |
| LanZhou Foci Pharmaceutical Co., Ltd. | 0.978 | 1.076 | 1.071 | 0.913 | 1.052 |
| Ginwa Enterprise (Group) Inc. | 0.928 | 1.053 | 0.967 | 0.96 | 0.977 |
| Yunnan Baiyao Group Co., Ltd. | 1 | 1.117 | 1 | 1 | 1.117 |
| PKU HealthCare Co., Ltd. | 0.984 | 1.061 | 1.036 | 0.951 | 1.044 |
| Chengdu huasun technology group Inc., Ltd. | 1.031 | 1.065 | 1.085 | 0.949 | 1.098 |
| Huapont Life Sciences Co., Ltd. | 1.079 | 1.094 | 1.016 | 1.063 | 1.181 |
| Tibet Cheezheng Tibetan Medicine Co., Ltd. | 0.993 | 1.081 | 1.019 | 0.974 | 1.074 |
| Guizhou Xinbang Pharmaceutical Co., Ltd. | 0.879 | 1.047 | 0.93 | 0.945 | 0.92 |
| Sichuan Kelun Pharmaceutical Co., Ltd. | 0.944 | 1.105 | 0.945 | 1 | 1.043 |
| Guizhou Bailing Group Pharmaceutical Co., Ltd. | 0.969 | 1.091 | 0.995 | 0.974 | 1.057 |
| Haisco Pharmaceutical Group Co., Ltd. | 1.015 | 1.058 | 1.009 | 1.006 | 1.074 |
| Kunming Longjin Pharmaceutical Co., Ltd. | 0.997 | 1.055 | 1.058 | 0.943 | 1.052 |
| Chengdu Kanghong Pharmaceutical Group Co., Ltd. | 0.892 | 1.043 | 0.871 | 1.024 | 0.931 |
| Chongqing Lummy Pharmaceutical Co., Ltd. | 1.007 | 1.029 | 0.998 | 1.009 | 1.036 |
| Chongqing Zhifei Biological Products Co., Ltd. | 1.22 | 1.058 | 1.206 | 1.011 | 1.291 |
| Walvax Biotechnology Co.,Ltd. | 1.226 | 1.033 | 1.186 | 1.034 | 1.266 |
| Fuan Pharmaceutical (Group) Co., Ltd. | 1.066 | 1.083 | 1.056 | 1.009 | 1.154 |
| Porton Pharma Solutions Ltd. | 0.965 | 1.082 | 0.949 | 1.017 | 1.044 |
| Sichuan Goldstone Asia Pharmaceutical Inc. | 0.924 | 1.094 | 1.003 | 0.921 | 1.011 |
| Maccura Biotechnology Co., Ltd. | 0.98 | 1.085 | 0.969 | 1.011 | 1.064 |
| Chongqing Taiji Industry (Group) Co., Ltd. | 0.876 | 1.074 | 0.879 | 0.997 | 0.941 |
| Tibet Rhodiola Pharmaceutical Holding Company. | 0.977 | 1.058 | 1.048 | 0.932 | 1.033 |
| Guizhou Salvage Pharmaceutical Co., Ltd. | 1.018 | 1.056 | 0.966 | 1.054 | 1.075 |
| KPC Pharmaceuticals, Inc. | 0.988 | 1.047 | 0.967 | 1.022 | 1.034 |
| Guizhou Yibai Pharmaceutical Co., Ltd. | 0.981 | 1.042 | 1.02 | 0.962 | 1.022 |
| Lionco Pharmaceutical Group Co., Ltd. | 1.009 | 1.078 | 1.07 | 0.943 | 1.087 |
| Average Value | 1.001 | 1.071 | 1.013 | 0.988 | 1.072 |
